# Multiscale Modeling of Macromolecular Interactions between Tau-Amylin Oligomers and Asymmetric Lipid Nanodomains That Link Alzheimer’s and Diabetic Diseases

**DOI:** 10.3390/molecules29030740

**Published:** 2024-02-05

**Authors:** Natalia Santos, Luthary Segura, Amber Lewis, Thuong Pham, Kwan H. Cheng

**Affiliations:** 1 Neuroscience Department, Trinity University, San Antonio, TX 78212, USA; nsantos@trinity.edu (N.S.); lsegura1@trinity.edu (L.S.); alewis2@trinity.edu (A.L.); 2 Physics Department, Trinity University, San Antonio, TX 78212, USA; tpham3@trinity.edu

**Keywords:** protein-lipid binding, anionic lipid nanodomains, protein folding, Alzheimer’s and diabetics crosstalk, amyloid-raft structures, lipid raft, neuronal membrane leaflets, oligomers

## Abstract

The molecular events of protein misfolding and self-aggregation of tau and amylin are associated with the progression of Alzheimer’s and diabetes, respectively. Recent studies suggest that tau and amylin can form hetero-tau-amylin oligomers. Those hetero-oligomers are more neurotoxic than homo-tau oligomers. So far, the detailed interactions between the hetero-oligomers and the neuronal membrane are unknown. Using multiscale MD simulations, the lipid binding and protein folding behaviors of hetero-oligomers on asymmetric lipid nanodomains or raft membranes were examined. Our raft membranes contain phase-separated phosphatidylcholine (PC), cholesterol, and anionic phosphatidylserine (PS) or ganglioside (GM1) in one leaflet of the lipid bilayer. The hetero-oligomers bound more strongly to the PS and GM1 than other lipids via the hydrophobic and hydrophilic interactions, respectively, in the raft membranes. The hetero-tetramer disrupted the acyl chain orders of both PC and PS in the PS-containing raft membrane, but only the GM1 in the GM1-containing raft membrane as effectively as the homo-tau-tetramer. We discovered that the alpha-helical content in the heterodimer was greater than the sum of alpha-helical contents from isolated tau and amylin monomers on both raft membranes, indicative of a synergetic effect of tau-amylin interactions in surface-induced protein folding. Our results provide new molecular insights into understanding the cross-talk between Alzheimer’s and diabetes.

## 1. Introduction

The uncontrolled aggregations of misfolded amyloidogenic proteins, e.g., the tau in the neurons of the brain and the amylin in the beta cells of the pancreas, have separately been linked to the early molecular events of the progression of Alzheimer’s (AZ) and type-2 diabetes (T2D), respectively [1,2,3]. Recent studies suggest that amylin and tau engage in cross-seeding to form hetero-tau-amylin oligomers [4,5,6], and those hetero-oligomers are more neurotoxic than the homo-tau aggregates alone [4,7]. Interestingly, an in vitro study indicates that the tau and amylin aggregate rapidly in solution at low concentrations. Furthermore, the protein secondary structures of the tau-amylin aggregates are not the same as those of the tau or amylin aggregates [8]. At present, the early macromolecular interactions of these hetero-amylin-tau aggregates on functionalized lipid nanodomains of the neuronal membrane that link to the early molecular cross-talk between AZ and T2D are unknown. 

The neuronal plasma membrane contains asymmetric, compartmentalized, and functionalized lipid nanodomains, or raft membranes, in both the inner and outer leaflets of the lipid bilayer [9,10]. Here, mono-unsaturated phosphatidylserine (PS) and ganglioside (GM1) are the major anionic lipid markers located exclusively in the inner and outer leaflets of the neuronal plasma membrane, respectively [10,11,12]. These anionic lipids play major physiological roles in signal transduction, protein docking, and protein sorting in the neurons [11,12]. In addition, recent computational and experimental studies indicate that PS-clusters and GM-clusters form functionalized nanodomains that regulate the membrane disruption and protein-folding activities of various homogeneous amyloidogenic protein aggregations from beta-amyloid, tau, and amylin on neuronal membranes [9,13,14,15,16]. These PS- and GM1-functionalized nanodomain interactions with early amyloid aggregates, or oligomers, may represent early protein-lipid interaction events on the inner and outer leaflets of the neurons that are associated with the membrane damage, toxicity, and death of the neurons [16]. So far, the detailed macromolecular interactions of hetero-tau-amylin aggregates with these leaflet-specific, functionalized lipid nanodomains of neurons await to be explored. 

Recently, we have designed and created two hetero-tau-amylin oligomers, a dimer and a tetramer, in solution from disordered tau and amylin monomers via a self-assembling approach based on the microsecond resolved coarse-grained (CG) molecular dynamics (MD) simulation [17]. In addition, we observed fast membrane binding of these hetero-oligomers to the lipid nanodomain membrane, or a CO-raft, containing saturated phosphatidylcholine (PC), unsaturated PC, and cholesterol [17]. However, the CO-raft presented in the previous work does not contain anionic lipids, which are important in regulating the membrane binding and protein folding behaviors of various membrane-active amyloid aggregates on lipid membranes [16]. In this study, we added a fourth lipid component, PS or GM1, to one lipid leaflet of the above CO-raft membrane. With the additional lipid component, we have created the asymmetric PS-raft or GM-raft, respectively [13,15]. Under physiological conditions, flexible and dynamic and phase-separated liquid-ordered (Lo) domains, liquid-disordered (Ld) domains, mixed Lo/Ld (Lod) domains, and PS- or GM1-clusters in the Lo domain are spontaneously formed on the microsecond time scale [13,15]. The PS- or GM1-clusters are located in only one leaflet of the bilayer, mimicking the functionalized lipid nanodomains exclusively found in the intracellular (inner) leaflet or extracellular (outer) leaflet of the neuronal membrane [9,11,12]. Here, with CG simulations, the macromolecular interactions of the hetero-oligomers with these PS- or GM1-functionalized lipid nanodomains were investigated. From the CG results, the microsecond-long lipid binding behaviors of hetero-oligomers were investigated. The kinetics of protein-lipid binding, protein binding patterns, and domain structural alterations were obtained. Other than the global lipid-protein interactions, localized disruptions of lipids structures and surface-induced protein folding were examined after a CG to all-atom (AA) spatial mapping [18] transformation, followed by a 100 ns-long AA MD simulation. Here, the binding or interaction energetics of protein-lipid and protein-protein interactions, lipid chain orientations, and protein secondary structures of the AA hetero-oligomers on raft surfaces were obtained.

Our multiscale MD simulations allow efficient sampling of the rotational and translational phase space of the microsecond-resolved protein-lipid binding events and exploring the nanosecond-resolved and atomistic membrane damage and protein behaviors of the protein on raft membrane surfaces. Our results suggest that PS- and GM-clusters are key membrane-binding sites of hetero-oligomers via hydrophobic and hydrophilic protein-lipid interactions, respectively. By comparing our results with the homo-tau and homo-amylin oligomers, the specific and global effects of membrane damage and surface-induced protein folding on the functionalized raft membranes involving tau and amylin were revealed. Similar to the homo-tau oligomers, the hetero-oligomers strongly disrupt the lipid orientational order of PS and GM1. Other than surface-induced beta-sheet formation upon protein binding to the raft membranes on both homo- and hetero-oligomers, we discovered an interesting synergetic role of amylin in promoting the alpha-helix formation of tau in the hetero-oligomer. The above molecular events of hetero- and homo-oligomer binding to functionalized lipid nanodomains provide new insights into revealing the intriguing molecular cross-talk between T2D and AZ [19]. 

## 2. Results

### 2.1. Lipid Binding Kinetics of Hetero-Oligomers 

We have modeled two pre-equilibrated and highly dynamic heterogeneous tau-amylin oligomers, a hetero-dimer (1tam) and a hetero-tetramer (2tam), in solution via a self-assembling process of equilibrated amylin and tau monomers based on the microsecond-resolved CG-MD simulations [17]. The primary sequences of the 37 residue-long-amylin and the 130-residue-long tau monomers, as well as their hydrophobicity profiles [14,15], are known and are summarized in Figure S1. Key hydrophobic residues of amylin are C7, L16, I26, and V32 (Figure S1B), and those of tau are V6, I35, I66, I86, I112, and V121 (Figure S1G). It is clear that the hydrophobic regions are much broader and better defined in amylin than in tau. The CG structures of 1tam and 2tam before, during, and after the self-assembling process in solution are further illustrated in Figure S2. The above hydrophobic residues of amylin and tau were randomly distributed in homo-oligomers (Figure S1) and hetero-oligomers (Figure S2). The average sizes of the hetero-dimer and tetramer were ~2 and 4 nm, respectively. The highly dynamic nature of hetero-oligomers in solution is also demonstrated in Videos S1 and S2 in Supplementary Materials. 

Three independent simulation replicates correspond to placing the oligomer at three different positions above the raft membrane surface before the simulations were created (see Section 4). With two hetero-oligomers: 1tam and 2tam, and two asymmetric raft membranes: PS-raft and GM-raft, a total of 12 independent, 15 μs-long-CG simulation replicates of hetero-oligomer/raft complexes were systematically investigated spanning 180 μs of CG-simulation time or ~720 μs of real-time based on the fact that CG water has ~4× faster diffusion than real water in CG simulations [20]. 

**Figure 1:**
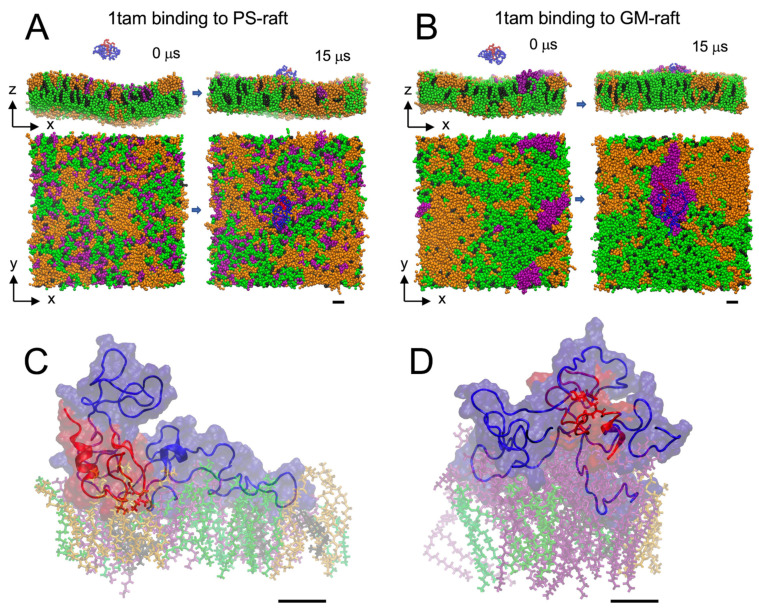
Modeling hetero-tau-amylin oligomer binding to raft membranes. The representative initial (0 μs) and final membrane-bound (15 μs) CG structures of 1tam/PS-raft (**A**) and 1tam/GM-raft (**B**) complexes from representative replicates in both transverse (*x*-*z*) and lateral (*x*-*y*) views are illustrated. The DPPC, DLPC, CHOL, and POPS (or GM1) lipids are shown in green, orange, black, and purple beads, and the protein structures are represented in backbone ribbon forms with chain A in blue and chain B in red. A CG-to-AA spatial transformation step converted the 15 μs-structures of CG 1tam/PS-raft and 1tam/GM-raft complexes to the corresponding AA structures. Thereafter, the 100 ns-long AA simulations were performed, and the representative final 100 ns-structures of 1tam/PS-raft (**C**) and 1tam/GM-raft (**D**) complexes are given. Other than backbone ribbons, colored AA protein surfaces of all protein atoms are shown. The AA lipids are represented in licorice with identical color assignments as the CG lipids. All CG and AA simulations were performed in 0.1 M NaCl and under physiological conditions of 1 atmosphere and 310 K. The two major hydrophobic residues at L16 and I26 of the amylin chain in the 1tam are shown in licorice. A scale bar of 1 nm is shown for each CG or AA protein/raft complex.

The microsecond binding behaviors of the hetero-dimer to the PS-raft and the GM-raft are demonstrated in Figure 1A,B, respectively. The modeling and contents of the PS-raft and GM-raft are described in Section 4. Both the transverse (*x*-*z*) and lateral (*x*-*y*) views of a representative hetero-dimer/raft complex before and after the 15 μs-long-CG simulations are shown in Figure 1. Here, the *z*-axis represents the normal of the lipid bilayer plane. We observed that the PS and GM1 lipids formed clusters within the DPPC-rich Lo domains in the top lipid leaflets of the PS-raft and GM-raft, respectively. This asymmetric distribution of PS- and GM1-clusters was preserved during the entire 15 μs CG simulation for all simulation replicates. In other words, no translocations of PS or GM1 lipids from one lipid leaflet to the opposite lipid leaflet were evident in our CG simulations. For all 12 CG replicates, each CG oligomer bound to the membrane surface within the first 8 μs and remained firmly attached to the membrane surface for the rest of the 15 μs simulation. No dissociations of the constituent monomers on the raft surfaces were also observed. Videos S3–S8 in Supplementary Materials demonstrate the lipid binding behaviors of the hetero-dimer to the CO-, PS-, and GM-rafts in both the transverse and lateral views from 0 to 15 μs. 

After the CG simulations, the equilibrated 15 μs-CG structure of each simulation replicate was transformed to an AA structure using a CG-to-AA spatial resolution transformation (see Section 4). Each transformed AA structure then underwent a 100 ns-long-AA simulation. Figure 1C,D demonstrate the equilibrated 100 ns-structures of the 1tam/PS-raft and 1tam/GM-raft complexes, respectively. For clarity, only the lipid molecules in the 0.5 nm annular lipid shell (see Section 4) are illustrated. Again, for all 12 replicates, each AA oligomer remained firmly attached to the membrane surface, and no dissociations of the constituent monomers during the entire 100 ns AA simulation were evident. 

To assess the lipid binding kinetics of the hetero-oligomers to the raft membranes, a minimum distance (mindist) statistical analysis (see Section 4) was performed on all 12 simulation replicates at both CG and AA resolutions.

The lipid binding kinetics of the hetero-dimer to the PS-raft were examined based on the calculated minimum distance between protein and lipid atoms vs. simulation time (upper panel) plots of all three replicates, as shown in Figure S3A–C. Here, large fluctuations of mindist values from 0–2, 0–8.2, and 0–1.8 μs followed by stable mindist values of ~0.5 nm were evident in the three independent replicates 1, 2, and 3, accordingly. The observation of large fluctuating mindist values in each replicate suggests that the protein stayed in the solution phase but made numerous transient contacts with the raft membrane surface. On the other hand, a stabilized mind indicates that the dimer is in a stable membrane-bound state. Here, the time of transition from fluctuating to stabilized mindist values at 2, 8.2, or 1.8 μs is defined as the lipid-binding time of the hetero-dimer to the PS-raft membrane for each replicate, accordingly. The number of contacts between protein and lipid atoms vs. time (mid-panel) plot was also used to examine the binding kinetics. Here, the number of contacts showed an abrupt transition from zero to large values indicative of an onset of the protein-lipid binding event, and the times of transition from the number of contacts vs. time plots were detected at 2, 8.2, and 1.8 μs and agreed with those from the mindist vs. time plots for the same replicates, accordingly. Similar observations of stable hetero-tetramer binding to the PS-raft membranes were also found (Figure S4A–C) among all three replicates. Here, the lipid-binding times were 1.8, 0.7, and 4.2 μs for replicates 1–3. Interestingly, the lipid-binding time for each replicate in the hetero-dimer/PS-raft or hetero-tetramer/PS-raft complex was the same for all lipid types, i.e., DPPC, DLPC, CHOL, and POPS. The results suggest that all four lipid types were responsible for initiating the microsecond-resolved lipid-binding event. 

The binding kinetics of the hetero-oligomers to the GM-raft membrane were also examined from the mindist perspective, as were the number of contacts vs. time plots. Again, stable oligomer binding to the raft membranes was evident for both hetero-dimers (Figure S5A–C) and hetero-tetramers (Figure S6A–C). Here, the GM1-binding times were 2, 0.2, and 1 μs for the hetero-dimer but 1.8, 4.0, and 0.1 μs for the hetero-tetramer for the protein-to-GM1 binding. However, some differences in the lipid-protein binding times for other lipid types were evident. Specifically, a few microseconds of time lag in the protein-lipid binding for non-GM1 lipids was clearly found in some replicates, e.g., replicate 1 (Figure S5A) and replicate 3 (Figure S5C) of the hetero-dimer, as well as replicate 2 (Figure S6B) and replicate 3 (Figure S6C) of the hetero-tetramer. In addition, we observed some transient behaviors of the protein-DLPC binding in replicate 3 of the hetero-tetramer/GM-raft complex, indicative of the intermittent association and dissociation of the hetero-tetramer with the DLPC. 

### 2.2. Lipid and Water Binding Patterns of Hetero-Oligomers

Both lipid and water binding patterns of hetero-oligomers in the raft membranes were determined by examining the time-averaged mindist vs. protein residue number plots, also defined as the mindist spectrum (see Section 4), over the stable membrane binding interval for each simulation replicate. The lower panels of Figures S3–S6 show the mindist spectra of all 12 replicates of the hetero-oligomer/raft complexes at both CG (panels A–C) and AA (panels D–F) spatial resolutions. The last 5 μs and 50 ns of the CG and AA simulations were used for the time-averaged calculation of the mindist spectra. Careful examinations of the mindist spectra revealed minima of protein-lipid and peaks of protein-water mindist spectra for both hetero-dimer and -tetramer in PS- and GM-raft membranes. The minimum mindist value for the AA simulations was ~0.2 nm, while that for the CG simulations was ~0.5 nm. Similar protein-lipid and protein-water mindist spectral features were observed in both AA and CG simulations. We focused our mind-based spectral analysis on the AA simulations in this study.

**Figure 2:**
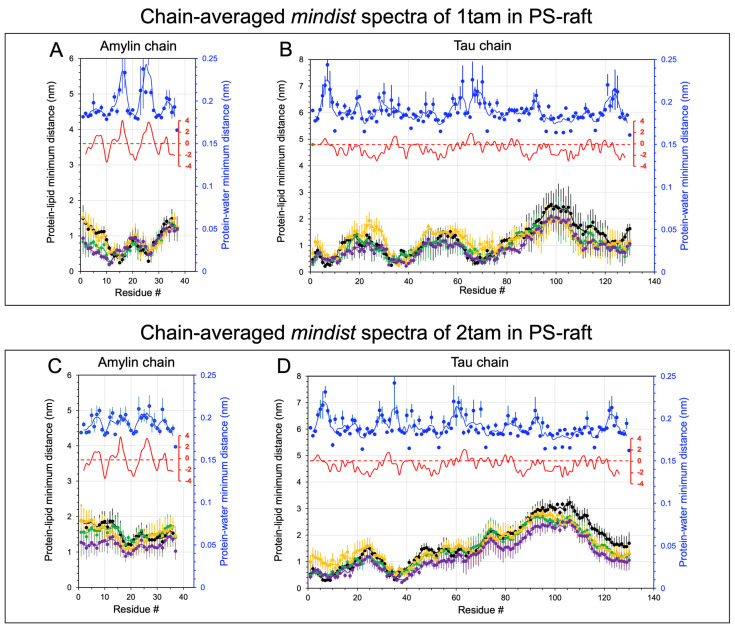
Minimum distance spectral analysis of hetero-tau-amylin oligomers in the PS-raft membrane. The minimum distance (*mindist*) spectrum, defined as time-, replicate-, and chain-averaged minimum distance between protein and lipid (or water) vs. protein residue, is shown for the 1tam/PS-raft (**A**,**B**) and 2tam/PS-raft (**C**,**D**) complexes. Each data point represents the average over the last 50 ns, across three replicates, and over the constituent chain, amylin (**A**,**C**) or tau (**B**,**D**). The error bar represents the standard error of the mean. All *mindist* values are color-coded, with DPPC in green, DLPC in orange, CHOL in black, POPS in purple, and water in blue. The hydrophobicity plot is given in red (see Section 4) to facilitate the identification of the hydrophobicity regions in each chain. A 5-point moving average fit is presented for the *mindist* spectral plots.

**Figure 3:**
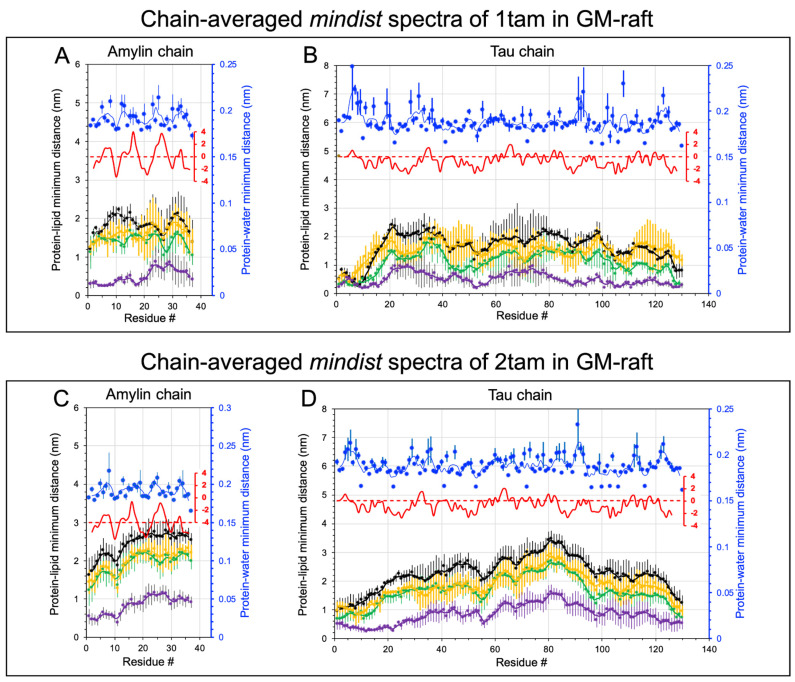
Minimum distance spectral analysis of hetero-tau-amylin oligomers in the GM-raft membrane. The minimum distance (*mindist*) spectrum, defined as time-, replicate-, and chain-averaged minimum distance between protein and lipid (or water) vs. protein residue, is shown for the 1tam/GM-raft (**A**,**B**) and 2tam/GM-raft (**C**,**D**) complexes. Each data point represents the average over the last 50 ns, across three replicates, and over the constituent chain, amylin (**A**,**C**) or tau (**B**,**D**). The error bar represents the standard error of the mean. The *mindist* values are color-coded, with DPPC in green, DLPC in orange, CHOL in black, GM1 in purple, and water in blue. The hydrophobicity plot is given in red (see Section 4) to facilitate the identification of the hydrophobicity residues in each chain. A 5-point moving average fit is presented for the *mindist* spectral plots.

Figure 2 and Figure 3 show the time- and replicate-averaged mindist spectra of the constituent amylin and tau chains inside the hetero-oligomer from the last 50 ns of the AA simulation and across three independent replicates of the hetero-dimer (panels A, B) and hetero-tetramer (panels C, D) in the PS-raft and GM-raft, respectively. Except for the amylin in the hetero-dimer (Figure 2A), the mindist values of the protein-PS or protein-GM1 were predominantly lower than those of protein-DPPC, -DLPC, and -CHOL for all hetero-oligomers in both raft membranes, indicating a preference of protein binding to the anionic PS or GM1 than to the neutral DPPC, DLPC, and CHOL, as shown in Figure 2B–D and Figure 3. For the amylin in hetero-dimer (Figure 2A), the mindist values of protein-DPPC and protein-PS were nearly identical, indicating an identical binding preference of amylin with either DPPC or PS. To facilitate the identification of the lipid binding patterns, we focused only on the protein-lipid mindist values below 1.0 nm, which is ~5× the lowest mindist value as described above. 

For the protein-lipid mindist spectra of hetero-dimer in the PS-raft, two minima at residues 12–15 and 23–26 of amylin for all lipid types (Figure 2A) and three minima at residues 5–15, 35–42, and 65–75 of tau for PS (Figure 2B) were evident. However, for the hetero-tetramer, only one minimum at residues 15–20 of amylin for PS (Figure 2C) and two minima at residues 5–15 and 35–42 of tau for PS (Figure 2D) were detected. Interestingly, prominent peaks in the protein-water mindist spectra at residues 25–26 of amylin (Figure 2A) and residues 6–8 of tau (Figure 2B) in the hetero-dimer, as well as residues 5–6 of tau (Figure 2D) in the hetero-tetramer, were found. These protein-water mindist spectral peak locations coincide with the protein-lipid mindist spectral minimum locations, indicative of the hydrophobic protein-lipid environments in those protein residues. Furthermore, those residue locations match the peak locations of the hydrophobicity vs. protein residue plot of each protein (see Figure S1) based solely on the known amino acid sequence of the protein. 

The lipid binding patterns of hetero-oligomers to the GM-raft were different from those to the PS-raft. For the hetero-dimer, a minimum baseline from residues 1 to 18 of amylin (Figure 3A) and two minima at residues 10–15 and 50–55, followed by a flat minimum baseline from residues 90–130 of tau (Figure 3B), were found. However, for the hetero-tetramer, a minimum baseline from residues 1 to 10 of amylin (Figure 3C) and two minima at residues 10–15 and 55 of tau (Figure 3D) were detected. Interestingly, those residue locations did not coincide with the peak locations of either the protein-water mindist spectra or hydrophobicity vs. residues plots of both amylin and tau, indicating that hydrophobic interactions are likely not involved in the protein binding to the GM-raft. 

### 2.3. Binding Energetics of Hetero- and Homo-Oligomers

**Figure 4:**
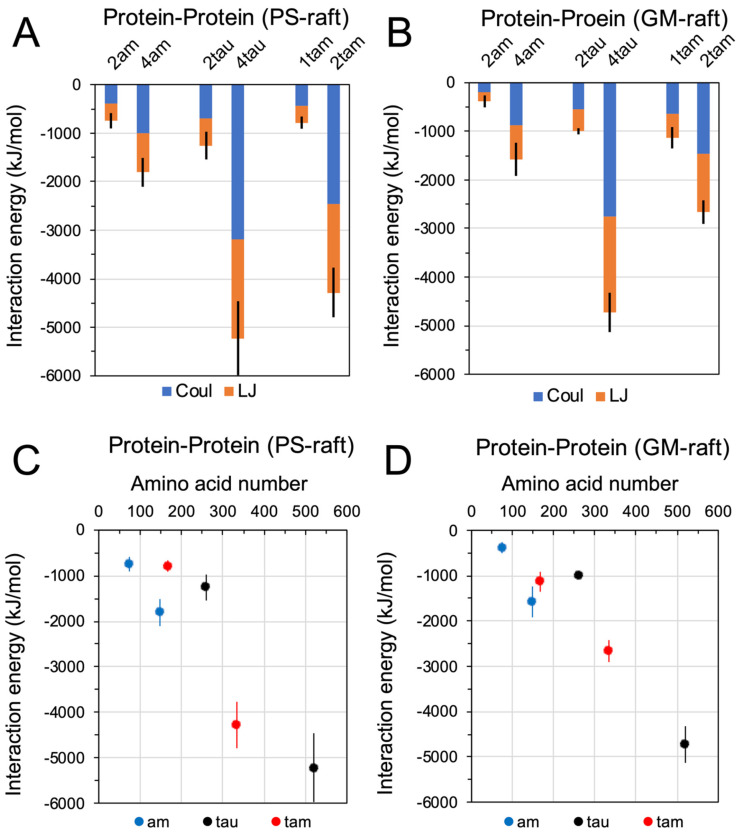
Protein-protein interactions of homo-oligomers and hetero-oligomers in the PS-raft and the GM-raft. The interaction energies among interchain protein atoms of homo-amylin (1am, 2am, and 4am), homo-tau (1tau, 2tau, and 4tau), and hetero-tau-amylin (1tam and 2tam) oligomers in the PS-raft (**A**,**C**) and the GM-raft (**B**,**D**) are given. The contributions of Coulomb (Coul) and Lennard Jones (LJ) energies are shown in a stacked histogram plot format (**A**,**B**). The total energy, i.e., the sum of Coul and LJ energies, of each oligomer is also presented in a scattered plot format (**C**,**D**), where the total amino acid number of each oligomer is given in the *x*-axis and the homo-amylin (blue), the homo-tau (black), and the hetero-tau-amylin (red) are color-coded. Each data point represents the time- and replicate-average over the last 50 ns and across all three independent replicates of the AA simulations. The error bar indicates the standard error of the mean.

**Figure 5:**
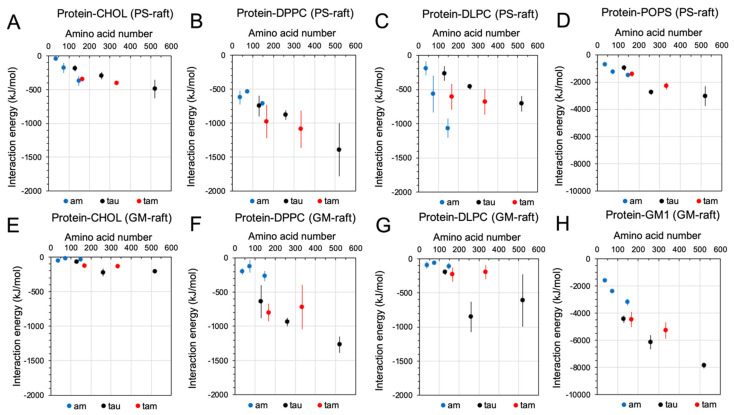
Protein-lipid interactions of homo- and hetero-oligomers in the PS-raft and the GM-raft. The interaction energies between protein and lipid atoms of homo-amylin (1am, 2am, and 4am), homo-tau (1tau, 2tau, and 4tau), and hetero-tau-amylin (1tam and 2tam) oligomers in the PS-raft (**A**–**D**) and the GM-raft (**E**–**H**) for CHOL (**A**,**E**), DPPC (**B**,**F**), DLPC (**C**,**G**), POPS (**D**), and GM1 (**H**) lipids are given. Each plot is presented in a scattered plot format where the total amino acid number of each oligomer is given in the *x*-axis, and the homo-amylin (blue), homo-tau (black), and hetero-tau-amylin (red) are color-coded. Each data point represents the time- and replicate average over the last 50 ns and across all three independent replicates of the AA simulations. The error bar indicates the standard error of the mean.

Protein-protein (Figure 4) and protein-lipid (Figure 5) interaction energies of the membrane-bound hetero- and homo-oligomers in the PS-raft and the GM-raft were systematically examined. 

Figure 4 shows the time- and replicate-averaged protein interchain interaction energies of hetero-oligomers, i.e., the dimeric 1tam and tetrameric 2tam, homo-amylin oligomers, i.e., the dimeric 2am and tetrameric 4am, and homo-tau-oligomers, i.e., the dimeric 2tau and tetrameric 4tau, from the last 50 ns of the AA simulations and across all three replicates of each oligomer. Both the non-covalent Lennard-Jones and Coulomb interactions (Figure 4A,B), as well as the sum of both (Figure 4C,D), were determined. Since each monomeric tau (1am) or monomeric amyloid (1tau) contains only one chain, no interchain interaction energy calculation was performed for those monomers. It is clear that both Lennard-Jones and Coulomb interactions contributed equally to the interchain interaction energy in all oligomers. 

To facilitate the comparison of protein-protein interaction energies of oligomers of different protein types, homo vs. hetero, and aggregation sizes, dimer vs. tetramer, a scattered plot of interaction energy vs. amino acid number of all 6 oligomers, differentiated by color-codes, in the PS-raft and GM-raft is presented in Figure 4C,D, respectively. Overall, the protein interchain energy increased linearly with the total amino acid number for all six oligomers of different types in both PS-raft and GM-raft. The protein interchain energy of a given oligomer in the PS-raft is similar to that in the GM-raft, with the exception of the 2tam. Here, the interchain energy of 2tam in the PS-raft was ~4000 kJ/mol, which was almost twice as large as that in the GM-raft. 

Figure 5 shows the time- and replicate-averaged protein-lipid interaction energies of 6 oligomers, also from the last 50 ns of the AA simulations and across all three replicates of each oligomer. The protein-lipid interactions between different lipid types were separately determined. In general, the protein-lipid interaction increased linearly with the total amino acid number for all lipid types, with the exception of the DLPC in both PS-raft (Figure 5C) and GM-raft (Figure 5G). For the PS-raft, a sharp increase in the protein-DLPC interaction from −200 to −1200 kJ/mol as the size of the homo-amylin-oligomer increased from monomer to tetramer was evident (Figure 5C). In contrast, only a moderate increase in the protein-DLPC interactions with the homo-tau-oligomer size was found. In addition, no significant differences in the protein-DLPC interaction of the 1tam and that of the 2tam were noticed. For the GM-raft, small protein-DLPC interactions around −100 to −300 kJ/mol for the homo-amylin and hetero-tau-amylin of all sizes, as well as the monomeric tau, were detected. However, the protein-DLPC energies of the dimeric and tetrameric tau oligomers were ~750 kJ/mol, which was much higher than that of the other oligomer. Among all lipid types, the protein-GM1 interaction energy (Figure 5H) was the highest, while the protein-CHOL interaction energy was the lowest. Overall, the protein-lipid interaction energies among different lipid types follow a ranking order of GM1 > POPS > DPPC ≈ DLPC > CHOL, particularly so for the tetramers. 

### 2.4. Disruptions of Lipid Domain Sizes by Hetero- and Homo-Oligomers 

**Figure 6:**
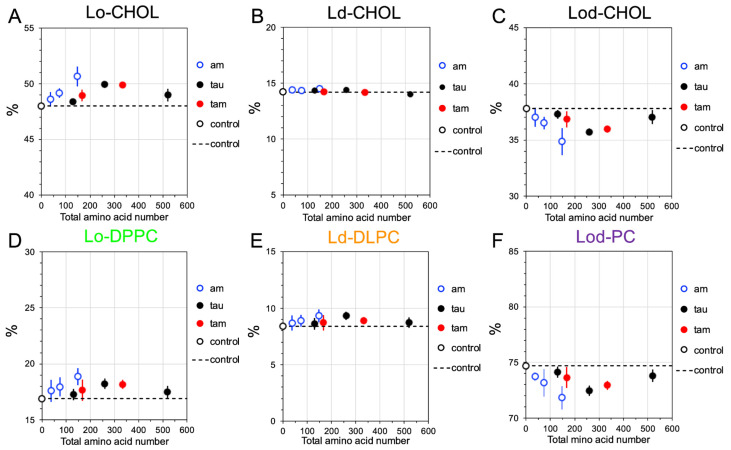
Lipid domain disruption by homo-oligomers and hetero-oligomers in the PS-raft. Plots of the percentages of CHOL in the Lo (**A**), Ld (**B**), and Lod (**C**) domains, and those of DPPC in the Lo domain (**D**), DLPC in the Ld domain (**E**), and the sum of DPPC and DLPC, or PC, in the Lod domain (**F**) vs. the total amino acid number of each oligomer are shown. Each percentage data point represents the time- and replicate-average over the time interval of stable membrane binding and across three independent replicates. Both homo-amylin oligomers (1am, 2am, and 4am), homo-tau oligomers (1tau, 2tau, and 4tau), and hetero-tau-amylin-oligomers (1tam and 2tam) are shown in blue circles, filled black circles, and filled red circles, respectively. The control at zero total amino acid number (open black circle) represents the percentage data without protein. The error bar indicates the standard error of the means.

**Figure 7:**
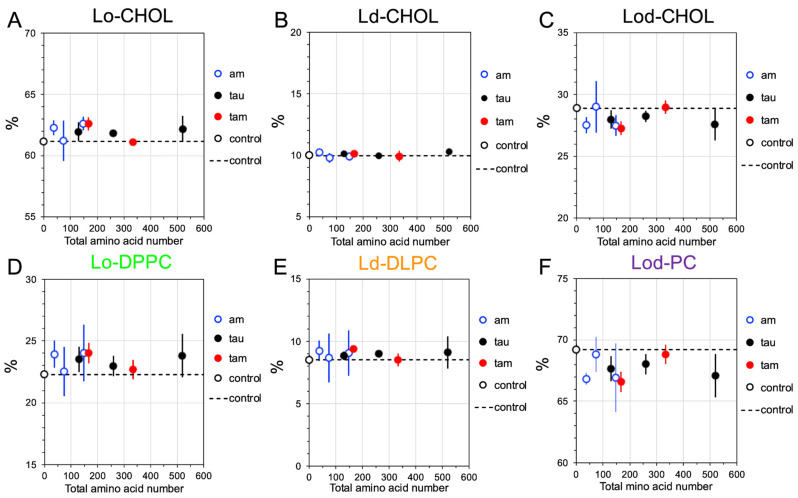
Lipid domain disruption by homo-oligomers and hetero-oligomers in the GM-raft. Plots of the percentages of CHOL in the Lo (**A**), Ld (**B**), and Lod (**C**) domains, and those of DPPC in the Lo domain (**D**), DLPC in the Ld domain (**E**), and the sum of DPPC and DLPC, or PC, in the Lod domain (**F**) vs. the total amino acid number of each oligomer are shown. Each percentage data point represents the time- and replicate-average over the time interval of stable membrane binding and across three independent replicates. Both homo-amylin oligomers (1am, 2am, and 4am), homo-tau oligomers (1tau, 2tau, and 4tau), and hetero-tau-amylin-oligomers (1tam and 2tam) are shown in blue circles, filled black circles, and filled red circles, respectively. The control at zero total amino acid number (open black circle) represents the percentage data without protein. The error bar indicates the standard error of the means.

To investigate the disruptive effects of the membrane-bound oligomers on the lipid domain sizes, the compositions of the Lo, Ld, and Lod domains from the last 5 μs of the CG simulations and across all three replicates of the raft membranes in the presence of and absence of hetero-oligomers (controls) were calculated. Here, a total of 8 oligomers of different types and sizes: 1am, 2am, 4am, 1tau, 2tau, 4tau, 1tam, and 2tam, were investigated. Figure 6 and Figure 7 show the time- and replicate-averaged compositions of the Lo, Ld, and Lod domains vs. the total amino acid number of each oligomer from the CG simulations in the PS-raft and GM-raft, respectively. As controls, the compositions of the lipid domains without oligomers are also given. Note that the sum of DPPC and DLPC% in the boundary Lod domain is labeled as Lod-PC (see Section 4).

For the PS-raft and in the absence of oligomers (Figure 6), the compositions of CHOL were ~48, 14, and 38%, and those of Lo-DPPC, Ld-DLPC, and Lod-PC were ~17, 8, and 75% in the Lo, Ld, and Lod domains, respectively. However, in the presence of membrane-bound oligomers, the values of CHOL% and PC% increased in the Lo domain (Figure 6A,D), remained unchanged in the Ld domain (Figure 6B,E), and decreased in the Lod domain (Figure 6C,F). A very similar domain-size disruption effect by oligomers was found for the GM-raft. Here, and in the absence of oligomers, the compositions of CHOL were ~61, 10, and 29%, and those of Lo-DPPC, Ld-DLPC, and Lod-PC were 22, 9, and 69% in the Lo, Ld, and Lod domains, respectively (Figure 7). In the presence of oligomers, an identical trend of CHOL and PC% changes in the presence of oligomers was evident when compared with the results for the PS-raft above. 

### 2.5. Disruptions of Lipid Acyl Chain Orientational Order by Hetero- and Homo-Oligomers

To examine the localized disruptions of the membrane-bound oligomers, the 0.5 nm annular lipids were extracted, and the lipid acyl chain orientational order profile, order parameter vs. carbon number, of these annular lipids was subsequently calculated (see Section 4). 

Figure S7 shows the time- and replicate-averaged numbers and percentages of DPPC, DLPC, CHOL, and POPS in the 0.5 nm annular lipid (AL) shell of the PS-raft over the last 50 ns of the AA simulations and across three independent replicates of the hetero- and homo-oligomer/raft complexes. We found that the number of lipids in the AL shell increased steadily with the amino acid number of the protein for all lipid types (Figure S7A–D). As a result, the percentages of CHOL, DPPC, DLPC, and POPS in the AL shell were ~30, 25, 15, and 30% for all oligomer types (Figure S7E–H). Similarly, Figure S8 shows the time- and replicate-averaged numbers and percentages of DPPC, DLPC, CHOL, and GM1 in the AL shell of the GM-raft. Again, a similar trend of an increase in the number of lipids in the AL shell with increasing amino acid numbers was found. However, the percentages of CHOL, DPPC, DLPC, and GM1 in the AL shell were ~15, 35, 15, and 35% for all oligomer types (Figure S7E–H), indicating a significant decrease in the CHOL and DPPC% in the GM-raft when compared with those in the PS-raft. 

Once the AL shells were identified, the disruptions of the orientational order of the lipid acyl chains vs. chain carbon number in the AL shells, or lipid order profiles, were investigated for all hetero-oligomer/raft complexes, and the results were compared with those for the homo-oligomer/raft complexes. Since each PC has two lipid acyl chains, i.e., *sn*-1 and *sn*-2 chains, the order profiles of both chains were calculated separately for all oligomers. As a control, the order profiles of the lipids outside the AL region (nAL) for each oligomer were also determined. 

**Figure 8:**
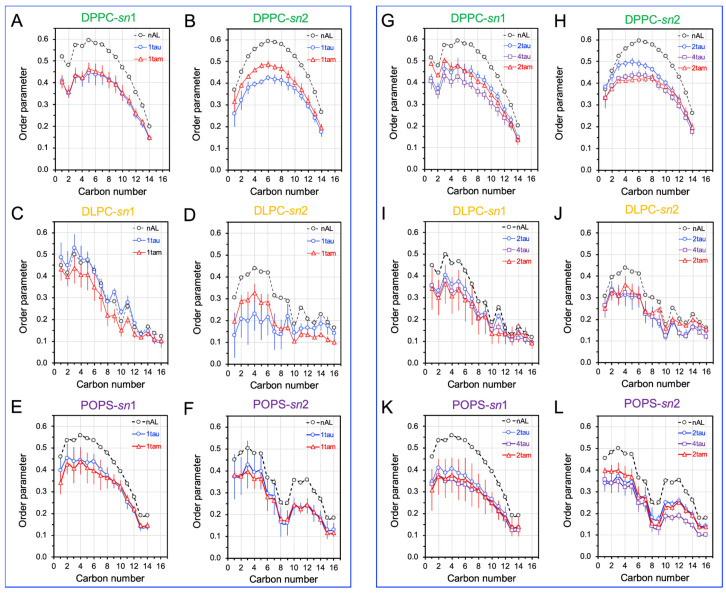
Phospholipid acyl chain order disruptions by homo-oligomers and hetero-oligomers in the PS-raft. The time- and replicate-averaged plots of the phospholipid orientational order parameter vs. lipid acyl chain carbon number, or lipid order profile, over the last 50 ns of the AA simulation and across all replicates for DPPC (**A**,**B**,**G**,**H**), DLPC (**C**,**D**,**I**,**J**), and POPS (**E**,**F**,**K**,**L**) lipids in the 0.5 nm annular lipid (AL) shell of the hetero-oligomers (1tam and 2tam) and homo-tau oligomers (1tau, 2tau, and 4tau) are shown. The lipid profiles of the lipids outside the AL shell, or non-annular lipids, are also given as controls. The data points for the *sn*-1 (**A**,**C**,**E**,**G**,**I**,**K**) and *sn*-2 (**B**,**D**,**F**,**H**,**J**,**L**) chains are shown. The error bar indicates the standard error of the mean.

The order profiles of DPPC, DLPC, and POPS in the AL shells of the PS-raft were examined, as shown in Figure 8. In general, the order profile of the *sn*-2 chain of either DPPC or DLPC was lower than that of the *sn*1-chain, as expected since the *sn*-2 chain is closer to the membrane surface than the *sn*-1 chain in a lipid bilayer. For the hetero-oligomers, the disruptions of the DPPCs *sn*-2 lipid order profile over the first 8 carbons by the tetrameric 2tam (Figure 8H) were much stronger than those by the dimeric 1tam (Figure 8B). No significant differences in lipid profile disruptions were evident in DLPC (Figure 8J vs. Figure 8D) or POPS (Figure 8L vs. Figure 8F).

**Figure 9:**
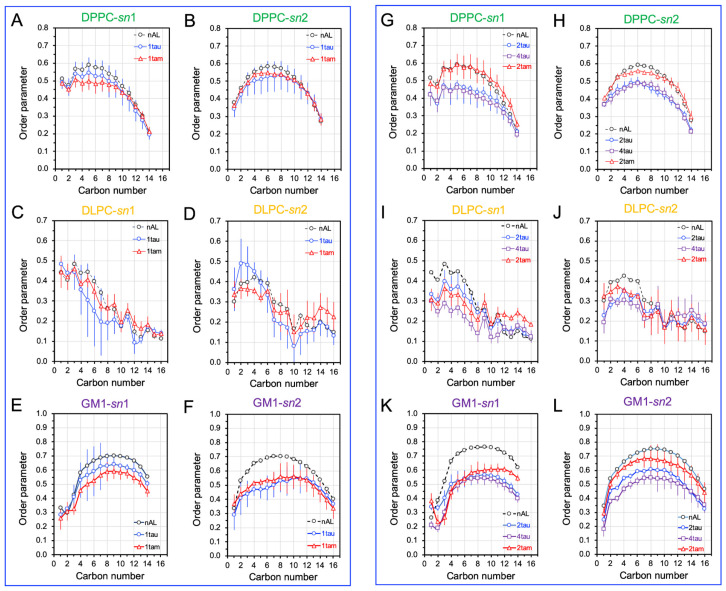
Phospholipid acyl chain order disruptions by homo-oligomers and hetero-oligomers in the GM-raft. The time- and replicate-averaged plots of the phospholipid orientational order parameter vs. lipid acyl chain carbon number, or lipid order profile, over the last 50 ns of the AA simulation and across all replicates for DPPC (**A**,**B**,**G**,**H**), DLPC (**C**,**D**,**I**,**J**), and GM1 (**E**,**F**,**K**,**L**) lipids in the 0.5 nm annular lipid (AL) shell of the hetero-oligomers (1tam and 2tam) and homo-tau oligomers (1tau, 2tau, and 4tau) are shown. The lipid profiles of the lipids outside the AL shell, or non-annular lipids, are also given as controls. The data points for the *sn*-1 (**A**,**C**,**E**,**G**,**I**,**K**) and *sn*-2 (**B**,**D**,**F**,**H**,**J**,**L**) chains are presented. The error bar indicates the standard error of the mean.

The order profiles of DPPC, DLPC, and GM1 in the AL shell of the GM-raft were also examined, as shown in Figure 9. For the hetero-oligomer, no significant disruptions of the lipid profiles of DPPC by either 1tam or 2tam were evident (Figure 9G,H vs. Figure 9A,B). However, a stronger disruption of the lipid profile of DLPC, particularly for the *sn*-1 chain, by the 2tam than by the 1tam was detected (Figure 9I,J vs. Figure 9C,D). Interestingly, the disruption of the lipid profile of the *sn*-2 chain of GM1 by the 1tam was significantly stronger than that by the 2tam (Figure 9F vs. Figure 9L). 

The disruptions of lipid profiles by the hetero-oligomers were compared with those by the homo-tau-oligomers of similar aggregation sizes in both PS-raft and GM-raft. For the DPPC in the PS-raft (Figure 8), the 1tau had a stronger disruptive effect than the 1tam, but the 2tam had a stronger disruptive effect than the 2tau (Figure 8B). In addition, the disruptive effect of the 2tam was stronger than that of the 2tau and even matched that of the 4tau (Figure 8H). No significant differences among hetero- and homo-tau oligomers in the disruptive effects were detected for the DLPC and POPS in the PS-raft. In the GM-raft, the disruptive effects of 1tam and 1tau were similar in all lipid types (Figure 8A–F). However, the 2tau and 4tau had a slightly stronger disruptive effect in both chains of DPPC than the 2tam (Figure 9G,H). Overall, similar but strong disruptive effects of homo-tau and hetero-oligomers were observed in the chains of GM1. 

### 2.6. Surface-Induced Protein Folding of Hetero- and Homo-Oligomers 

**Figure 10:**
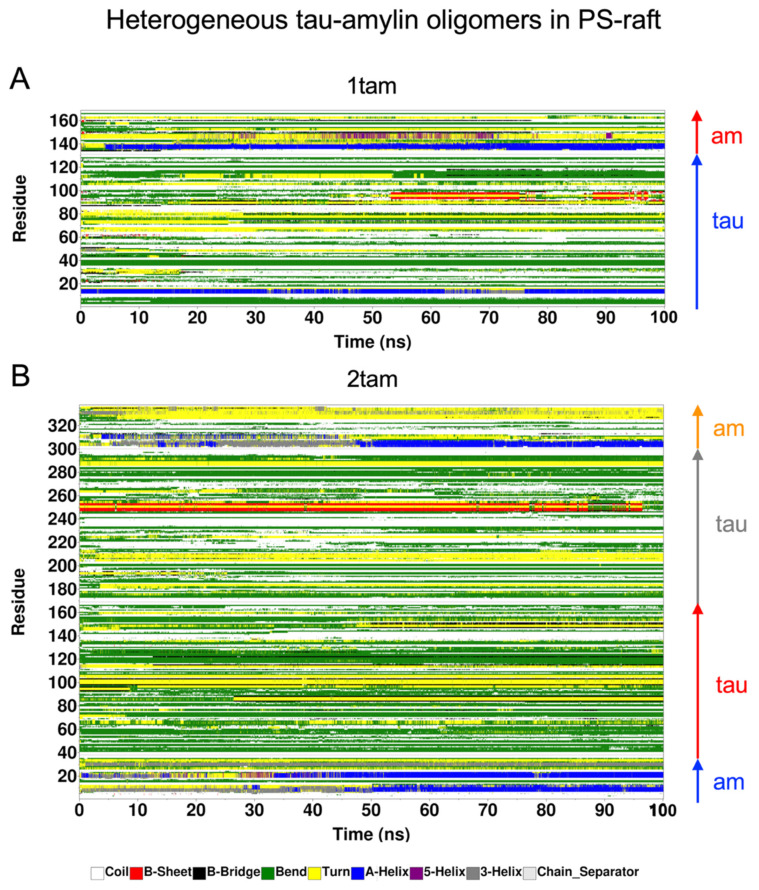
Time-evolution of protein secondary structures of membrane-bound hetero-oligomers upon binding to the PS-raft. The 3D color-coded protein secondary structures as a function of residue number (vertical axis) and simulation time (horizontal axis) are given in a DSSP format (see Section 4) for the representative replicates of the 1tam and 2tam oligomers. The protein residue locations of the tau and amylin (am) chains inside the hetero-oligomers are color-coded. For 1tam (**A**), the chain A (tau) and the chain B (amylin) are shown in blue and red arrows, respectively. For the 2tam (**B**), the chain A (am), the chain B (tau), the chain C (tau), and the chain D (am) are shown in blue, red, gray, and orange arrows, respectively.

**Figure 11:**
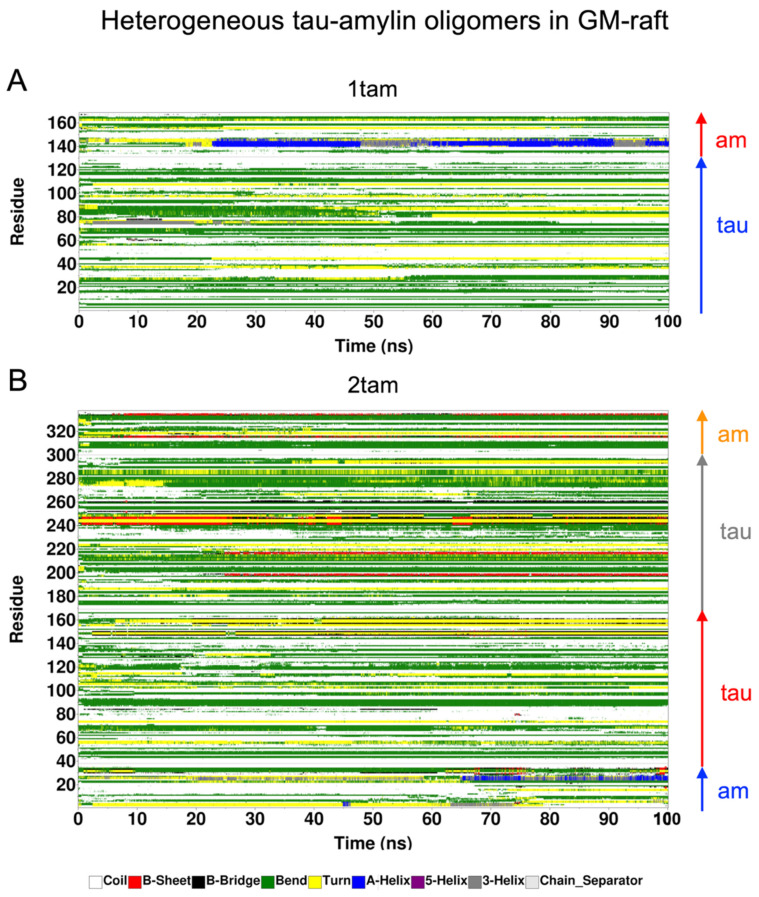
Time-evolution of protein secondary structures of membrane-bound hetero-oligomers upon binding to the GM-raft The 3D color-coded protein secondary structures as a function of residue number (vertical axis) and simulation time (horizontal axis) are given in a DSSP format (see Section 4) for the representative replicates of the 1tam and 2tam oligomers. The protein residue locations of the tau and amylin (am) chains inside the hetero-oligomers are color-coded. For 1tam (**A**), the chain A (tau) and the chain B (amylin) are shown in blue and red arrows, respectively. For the 2tam (**B**), the chain A (am), the chain B (tau), the chain C (tau), and the chain D (am) are shown in blue, red, gray, and orange arrows, respectively.

The time- and residue-resolved protein secondary structures, or DSSP profiles (see Section 4), of the membrane-bound hetero-oligomers in the PS-raft and GM-raft were examined as demonstrated in Figure 10 and Figure 11, respectively. The alpha-helices and beta-sheets represented the predominant protein secondary structures of the constituent amylin and tau chains inside the 1tam or 2tam bound to the PS-raft (Figure 10), respectively. Similar observations were found for the case of GM-raft, but detectable beta-sheets started to form in the amylin in the 2tam, as illustrated in Figure 11B. The DSSP profiles of all simulation replicates of hetero-oligomers are given in Figure S9. 

To streamline our protein folding analysis, some of the resolved secondary structures from DSSP were grouped into three newly defined structures: beta, alpha, and random. Here, the beta structure includes beta-sheets and -bridges; the alpha structure includes alpha-, 5-, and 3-helices; the random structure includes coils and bends; and the turn structure remains an independent structure. Note that the residues with the random structure do not involve hydrogen bonding, but the other structures do (see Section 4). 

**Figure 12:**
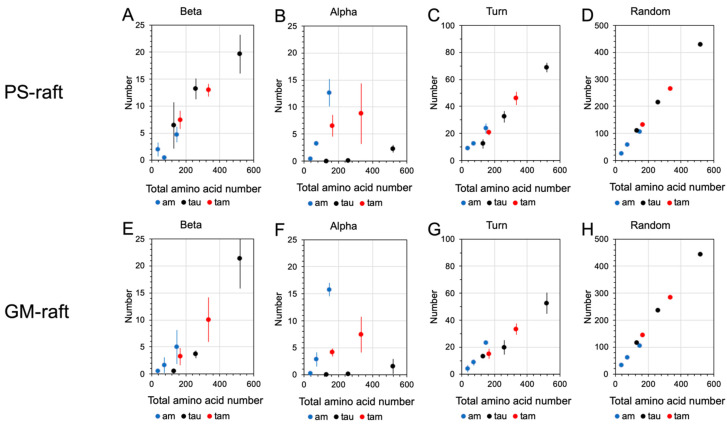
Protein secondary structures of membrane-bound homo-oligomers and hetero-oligomers in the PS-raft and GM-raft. The numbers of protein residues of the oligomers in the PS-raft (**A**–**D**) and the GM-raft (**E**–**H**) that participated in the beta (**A**,**E**), alpha-helix (**B**,**F**), turn (**C**,**G**), and random (**D**,**H**) structures are shown in a scattered-plot format where the total amino acid number of each oligomer is given in the *x*-axis and the homo-amylin (blue), homo-tau (black), and hetero-tau-amylin (red) are labeled in colors. Each data point represents the time- and replicate-average over the last 50 ns and across all three independent replicates of the AA simulations. The error bar indicates the standard error of the mean.

Figure 12 shows the averaged number of residues with the four reclassified secondary structures in the hetero- and homo-oligomers bound to the PS- and GM-rafts. Here, each data point represents the time- and replicate average over the last 50 ns of the AA simulation and across three simulation replicates of each membrane-bound oligomer. Interestingly, we discovered that the number of beta, turn, or random structures increased linearly with the total amino acid number for all oligomers in both the PS-raft (Figure 12A,C,D) and the GM-raft (Figure 12E,G,H). In contrast, a non-linear relationship between the number of alpha structures and the total amino acid number was evident for all oligomers in both raft membranes, as shown in Figure 12B,F. Specifically, in all raft membranes, we observed that the 4am had the largest number of alpha structures, and the tau oligomers of all sizes had the smallest number of alpha structures when compared with other homo- and hetero-oligomers. 

**Figure 13:**
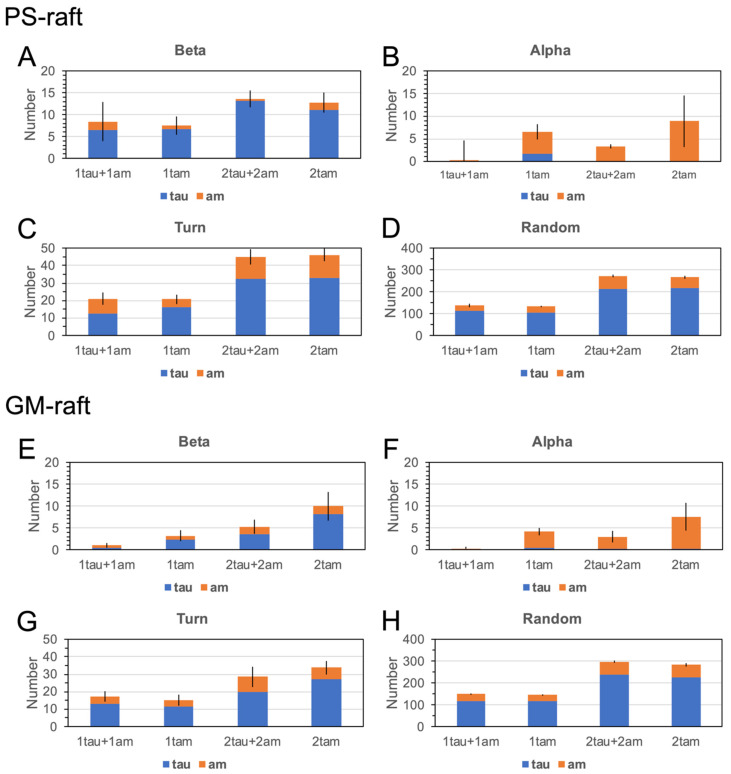
Protein secondary structures of the constituent proteins in membrane-bound homo-oligomers and hetero-oligomers. The number of residues in the PS-raft (**A**–**D**) and GM-raft (**E**–**H**) membranes that participated in the beta (**A**,**E**), alpha (**B**,**F**), turn (**C**,**G**), and random (**D**,**H**) structures is shown in a stacked histogram format. For the homo-oligomers, the sum of secondary structures from 1tau to 1am (1tau+1am) or the sum of 2tau and 2am (2tau+2am) group is directly compared alongside the secondary structure of 1tam or 2tam. Note that the total amino acid number of the (1tau+1am) or (2tau+2am) group is 167 or 334, respectively, and matches that of the 1tam or 2tam, accordingly. Each data point represents the time- and replicate-average over the last 50 ns and across all three independent replicates of the AA simulations. The error bar indicates the standard error of the mean.

**Figure 14:**
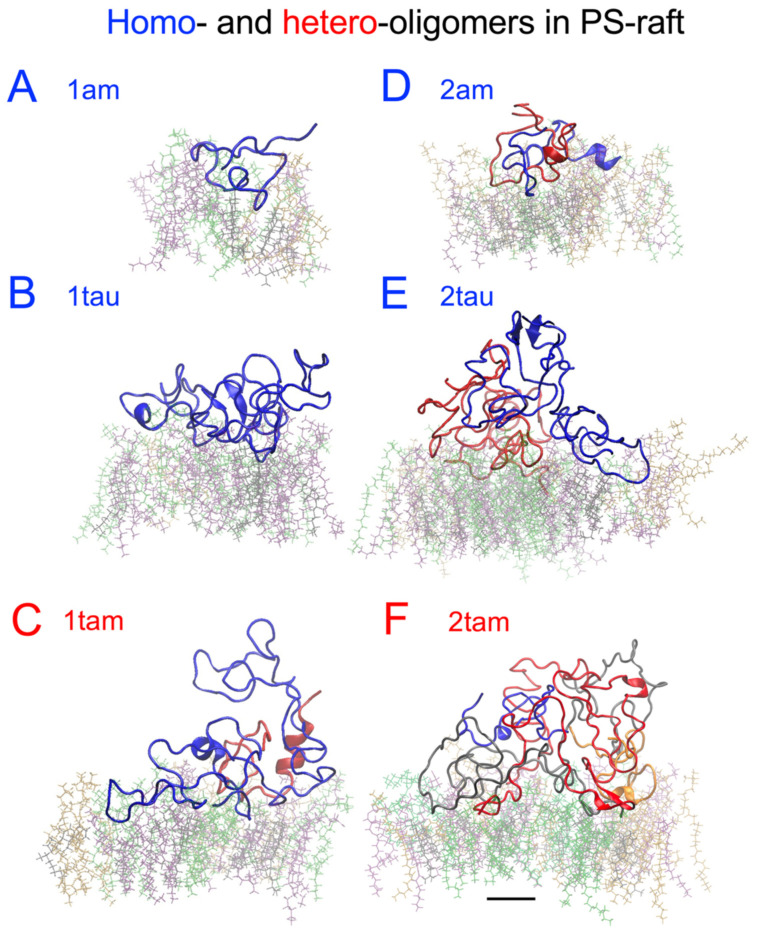
Structures of the membrane-bound homo-oligomers and hetero-oligomers are surrounded by the 0.5 nm annular lipid shell in the PS-raft. The secondary structures of the homo-amylin monomer or 1am (**A**), the homo-tau monomer or 1tau (**B**), the hetero-tau-amylin dimer or 1tam (**C**), the homo-amylin-dimer or 2am (**D**), the homo-tau dimer or 2tau (**E**), and the hetero-tau-amylin tetramer or 2tam (**F**) are shown. All protein structures are presented in color-coded backbone ribbons. For the homo-oligomers, chains A and B are labeled in blue and red, respectively. For the 1tam (**C**), chain A (tau) and chain B (am) are labeled in blue and red, respectively. For the 2tam (**F**), chain A (am), chain B (tau), chain C (tau), and chain D (am) are labeled in blue, red, gray, and orange, respectively. The 0.5 nm annular lipids within are color-coded in licorice, with DPPC in green, DLPC in orange, cholesterol in black, and POPS in purple. A scale bar of 1 nm is shown.

**Figure 15:**
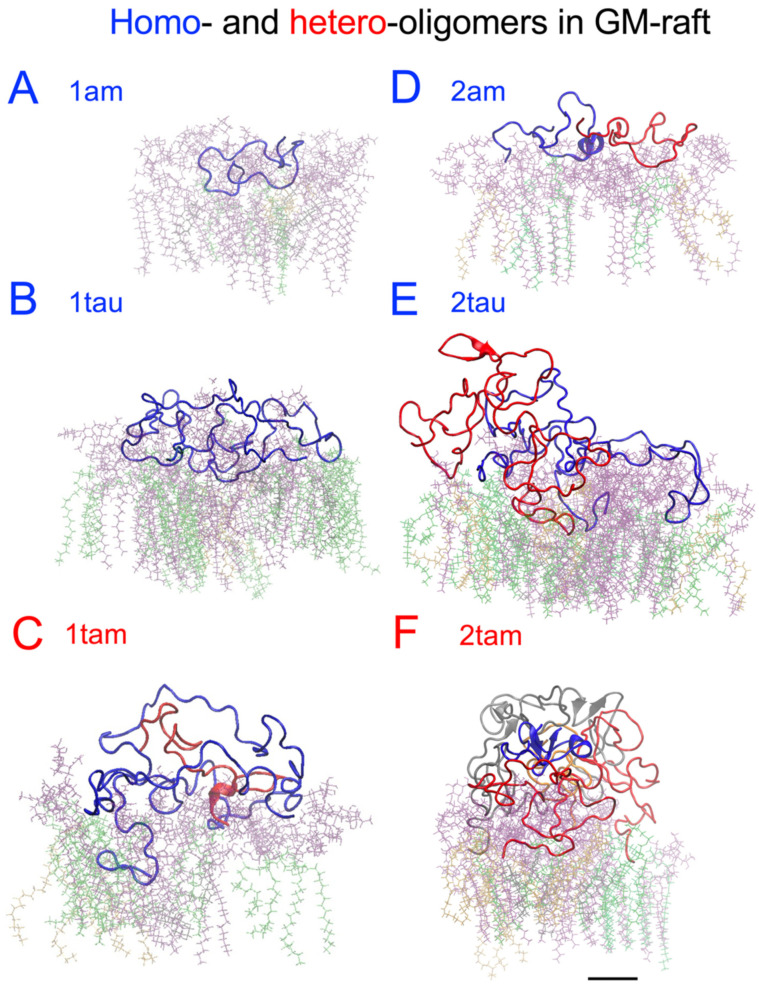
Structures of the membrane-bound homo-oligomers and hetero-oligomers surrounded by the 0.5 nm annular lipid shell in the GM-raft. The secondary structures of the homo-amylin monomer or 1am (**A**), the homo-tau monomer or 1tau (**B**), the hetero-tau-amylin dimer or 1tam (**C**), the homo-amylin-dimer or 2am (**D**), the homo-tau dimer or 2tau (**E**), and the hetero-tau-amylin tetramer or 2tam (**F**) are shown. All protein structures are presented in color-coded backbone ribbons. For the homo-oligomers, chains A and B are labeled in blue and red, respectively. For the 1tam (**C**), chain A (tau) and chain B (am) are labeled in blue and red, respectively. For the 2tam (**F**), chain A (am), chain B (tau), chain C (tau), and chain D (am) are labeled in blue, red, gray, and orange, respectively. The 0.5 nm annular lipids within are color-coded in licorice, with DPPC in green, DLPC in orange, cholesterol in black, and GM1 in purple. A scale bar of 1 nm is shown.

To assess the general effects between tau and amylin in protein folding on different raft surfaces, we directly compared the numbers of beta (Figure 13A,E), alpha (Figure 13B,F), turn (Figure 13C,G), and random (Figure 13D,H) structures of tau and amylin in the homo-oligomers with those of the constituent tau and amylin in the hetero-oligomers separately in a stacked-histogram format. Here, the sum of secondary structures of the homo-oligomer, i.e., 1tau+1am or 2tau+2am, is plotted alongside that of the hetero-oligomer, i.e., 1tam or 2tam, for tau and amylin, separately. Note that the total amino acid number of 1tau+1am is 167, which is identical to that of 1tam. Similarly, the total amino acid number of 2tau+2am is 334, again identical to that of 2tam. The representative secondary structures of amylin and tau in both homo- and hetero-oligomers and the corresponding AL lipid shells are also demonstrated in Figure 14 and Figure 15 for the PS-raft and the GM-raft, respectively. ANOVA analyses comparing the effects of protein and surface on beta structure formation revealed no significant differences across all types of oligomers. However, strong protein but not surface effects on the alpha-helix formation among several oligomers were detected, and the results are summarized in Table S1. Here, the alpha-helical content of 1tam was greater than that of 1tam+1tau (*p* < 0.01) but much greater than that of 2tau (*p* < 0.001). 

To further investigate the specific effects of tau-amylin interactions on the surface-induced protein folding for different protein and membrane types, the secondary structure contents of the constituent amylin monomer (1am’) and the constituent amylin dimer (2am’) in the dimeric 1tam, as well as those of the constituent tau monomer (1tau’) and the constituent tau dimer (2tau’) in the tetrameric 2tam, were extracted. These extracted secondary structures of the constituent monomers or dimers allow us to compare them with those of the monomers or dimers of the homo-tau and homo-amylin oligomers. Careful statistical assessments of the differences among the monomers and dimers from the hetero- and homo-oligomers are described below. 

We have conducted multiple ANOVA statistical analyses to investigate the effects of protein and raft surface types on alpha and beta structure formation. Figure S10 shows the effect of amylin on the folding of tau. Our results of comparing 1tau’ vs. 1tau revealed a marginal significance of *p* = 0.05 with preferential beta-folding for oligomers bound to the GM-raft instead of the PS-raft (Figure S10B). No significant effects of protein or raft surface were found for the alpha-helical structures. Figure S11 shows the effect of tau on the folding of amylin. Our results show that 1am’ formed significantly more alpha helices than 1am with *p* < 0.0001 (Table S1) on both PS-raft (Figure S11A) and GM-raft (Figure S11B). However, the comparison between larger amylin structures, i.e., 2am’ and 2am, did not show significant differences in alpha-helix formation (*p* > 0.05). No significant effects of tau on the beta-folding of amylin were evident (Figure 11B,D). 

### 2.7. Residue-Resolved Protein-Protein Contact Map

Based on a molecular interaction analysis tool, CONAN (see Section 4), the residue-resolved protein-protein interactions at the contacting interface between the constituent amylin and tau in the hetero-oligomers were examined using the contact maps. Figure S12 shows the contact maps of 1tam and 2tam in solution, and Figures S13–S16 show the contact maps of the membrane-bound 1tam and 2tam in CO-raft (no PS or GM1), PS-raft, and GM-raft, respectively. Overall, the results provide evidence that the amylin and tau monomers were strongly coupled in both solution- and membrane-bound states. The *C*-terminal of the amylin strongly interacted with mostly the middle region of tau in both solution- and membrane-bound states for the CO-raft and PS-raft. However, for the GM-raft, a strong contact between the *N*-terminal and tau, particularly the 1tau (Figure S15A), was found. In general, both the intra- and inter-chain protein-protein contacts were highly dynamic in both solution-bound and membrane-bound states, as clearly demonstrated in the standard deviation maps.

## 3. Discussion

Based on multiscale MD simulations, we have investigated the protein-lipid binding behaviors, protein-induced membrane disruptions, and surface-induced protein folding of the hetero-oligomers vs. homo-oligomers on the asymmetric raft membranes containing PS- and GM1-clusters. Those PS- and GM1-clusters mimic the anionic lipid nanodomains found in the intracellular and extracellular leaflets of the neuronal membrane [9,11,12,16], and our results, therefore, provide new information on the early molecular events of protein-lipid interactions of tau-amylin aggregations on both leaflets of the neuronal plasma membrane. 

Among the Lo, Ld, and Lod nanodomains, the hetero-oligomers preferentially bind to the Lod domain, as revealed by the mindist and AL shell composition analyses for both PS- and GM-rafts. This finding is in line with the recent computational studies on homo-tau [13] and homo-amylin [15] oligomers binding to the PS-raft and GM-raft. Interestingly, other cytotoxic and membrane-active proteins, such as the HIV-gp42 fusion protein and certain antimicrobial peptides [21,22,23,24,25,26], are also known to bind to the Lod domain. It is believed that the line tension at the domain boundaries due to the lipid bilayer thickness mismatch between the thicker Lo domain and the thinner Ld domain drives the membrane-active proteins to the dynamic Lod region [25,27,28,29]. Therefore, the boundary Lod domain may be a common lipid nanodomain region for other non-amyloidogenic and amyloidogenic proteins to bind to. We further propose that the Lod domain may represent a unique self-assembled macromolecular target for future drug interventions that aim to prevent toxic amylin, tau, and amylin-tau oligomers attachment to the host cell membranes in T2D [3], AZ [1], and the cross-talk between T2D and AZ [4,5,6,19], accordingly.

We discovered that the protein binding patterns of the hetero-oligomer to the PS-raft were different from those to the GM-raft. Here, regions around the hydrophobic L16 and I26 of amylin and V6 and I36 of tau were major membrane anchoring sites of both 1tam and 2tam to the PS-raft. In contrast, the *N*-terminals of amylin (residues 1–10) and tau (residues 1–15) were membrane anchoring sites of 1tam to the GM-raft. Additional membrane anchoring sites around residues 50–55 and the *C*-terminal (residues 100–130) of 2tam to the GM-raft were evident. In the case of GM-raft, most of the membrane anchoring regions are associated with the hydrophilic or polar residues for both amylin and tau. Therefore, we propose that the protein-lipid interactions between the protein residues of hetero-oligomers and the lipids are mainly hydrophobic for the PS-raft but mostly hydrophilic for the GM-raft. 

A linear correlation between the non-covalent or inter-chain protein-protein binding energy and the total amino acid number for the membrane-bound oligomers of different types and sizes was evident in both raft membranes. This result suggests that the aggregation energy of amyloidogenic oligomers increases with the number of amino acids in the amyloid peptides, independent of the protein type, or primary sequence, of the constituent peptides. Furthermore, a linear correlation between the protein-lipid binding energy and the amino acid number was also evident for all lipid types except DLPC in both PS- and GM-raft. Here, the macromolecular interactions between the highly unsaturated DLPC and the membrane-bound oligomer depend strongly on the protein type and the membrane composition. Specifically, the AL-DLPC lipids in the PS-raft bind stronger to the tetrameric amylin oligomer on the PS-raft than the other oligomers. On the other hand, the AL-DLPC lipids in the GM-raft bind stronger to the dimeric and tetramer tau oligomers than the other oligomers. The specific protein-type and raft-composition dependence on oligomer/DLPC suggests a key role of unsaturated lipids in protein-lipid binding energetics that may have important implications for the structure-function relationship of oligomer-raft interactions in cell membranes. Finally, a direct comparison of protein-lipid binding energies across oligomers of different types reveals a ranking order of GM1 > PS > DPPC ≈ DLPC > CHOL. Note that GM1, PS, and saturated DPPC represent the major lipid types found in the phase-separated lipid raft domain, and unsaturated DLPC is the major lipid type in the non-raft Ld and phase boundary Lod [9,30,31,32]. The above ranking suggests that all oligomers preferentially interact with the raft-associated lipids over the non-raft lipids. Therefore, raft lipids may represent the major membrane-attack targets in both homo- and hetero-oligomers. The cholesterol molecule is embedded below the polar surface of the raft membranes. The CHOL binding affinity of oligomers of all types, particularly the larger ones, was stronger in the PS-raft than the GM-raft. Since GM1 has a much larger polar carbohydrate headgroup than PS or PC, the difference in CHOL binding affinity is mainly attributed to the accessibility of the oligomer to the CHOL due to the thicker carbohydrate cushion separating the protein and the CHOL molecules in the GM-raft. 

Note that our protein-protein and protein-lipid binding energies, as described in Figure 4, are total interaction or potential energies among various interacting atoms within the 1.2 nm energy sampling threshold using the GROMACS energy tool (see Section 4). These binding energies depend on the number of interaction atoms in the protein and lipid groups. Future work will include the use of the Molecular Mechanics/Poisson-Boltzmann Surface Area approach to investigate the free energy of binding in the presence of solvents to obtain more detailed interactions among molecules in our membrane-bound hetero-oligomer complexes on different membrane surfaces. 

Note that recent computational investigations involving other hetero-oligomers containing beta-amyloid and amylin in solution [33] and on membrane surfaces [34] have been performed. Interestingly, our observation of the stronger interaction of our hetero-tau-amylin oligomers with the anionic lipid, i.e., GM1 or PS, than the neutral PC lipid agrees with the previous work on a hetero-oligomer containing beta-amyloid and amylin interacting with the PC and PC/PG membranes. Here, PG, or phosphatidylglycerol, is also an anionic lipid. Therefore, the hetero-oligomer/anionic lipid interaction may be a universal feature involving cross-seeded oligomers and lipid membranes. 

The extent of alteration of lipid domain compositions by membrane-bound oligomers was used to evaluate the oligomer-induced perturbation of the lateral organization of raft membranes. Here, we detected a small yet significant increase in the Lo-CHOL and Lo-DPPC contents in the Lo domain and a concomitant decrease in the Lod-CHOL and Lod-PC in the Lod domain in the presence of oligomers for both PS- and GM-rafts. These observations suggest that the oligomers exclusively affect the size of the Lo or Lod domain but not the size of the Ld domain in both raft membranes. Interestingly, the domain size perturbation effect is slightly stronger in the PS-raft than in the GM-raft.

We observed that the localized perturbation effects of oligomers on the lipid order profile, or the oligomer-induced membrane damaging effects, depend on the raft membrane type (PS-raft vs. GM-raft), lipid type, as well as the type (homo vs. hetero) and size (dimer vs. tetramer) of the oligomer. 

For the PS-raft, the hetero-dimer has a smaller perturbation effect than the monomeric tau in the order profile of DPPC, indicating that the presence of amylin in the hetero-dimer suppresses the perturbation effect of monomeric tau. In contrast, the presence of amylin in the hetero-tetramer has a much stronger perturbation effect on DPPC than the dimeric tau. In other words, the presence of amylin in the hetero-tetramer enhances rather than suppresses the perturbation effect of dimeric tau on DPPC. Our results indicate that hetero-tetramers are more structurally disruptive than homo-tau oligomers of similar sizes. Comparatively, no significant differences were found in the strong perturbation effects of the order profiles of DLPC and PS. Hence, this study identifies the strongest DPPC disruptive effect of hetero-tetramers in the PS-raft membrane when compared with homo-oligomers of similar sizes. 

The membrane-disruptive effects in the GM-raft are quite different from those in the PS-raft. Here, the hetero-oligomers of all sizes disrupted GM1 as effectively as the homo-oligomers. Yet no significant order disruption effects by hetero-oligomers on other lipid types were found. 

Recent in vivo studies have provided strong evidence that amylin exacerbates the cytotoxicity of tau [5,6,19]. Interestingly, we found that monomeric amylin forms an increased number of alpha-helical structures upon its interaction with tau in the hetero-oligomer, in comparison to monomeric amylin on raft membranes. Since the formation of the alpha structure is an important intermediate structure before the beta structure, we propose that the presence of amylin promotes the random-to-alpha-beta folding pathway of tau upon binding to the neuronal plasma membranes. The presence of both alpha-helical and beta-sheet structures in the membrane-bound homo- and hetero-oligomers on both raft membranes suggests that both secondary structures may participate in the formation of alpha- and beta-barrel transmembrane pores, as proposed in recent computational and experimental studies [16,35,36]. In addition, the prevalence of beta-sheet structures in all homo- and hetero-oligomers further suggests that these localized beta-sheets may act as a nucleation seed to recruit other amyloidogenic oligomers to create surface-bound fibrils [16]. Finally, recent studies strongly supported surface-induced fibril growth as another major membrane damage mechanism other than the creation of transmembrane pores by oligomers [16,35]. 

Finally, the strong binding effects of the hetero-oligomers to the GM1-clusters suggest that the preformed tau and amylin outside the neurons can readily interact with the neuron extracellularly. Therefore, the site of membrane attack by tau, amylin, and tau-amylin can occur both inside and outside the neurons. The atomistic structures of these membrane-bound oligomers will be useful for future therapeutic interventions that target those membrane-bound oligomer-PS and -GM1 complexes to prevent the early progression of Alzheimer’s and, importantly, the cross-talk between Alzheimer’s and diabetics. 

## 4. Materials and Methods

### 4.1. Modeling Hetero-Oligomers in Solution

Highly dynamic and equilibrated hetero-oligomers in solution were created via a self-assembling CG simulation process, as described in a recent study [17]. Starting from separated monomers, the design and modeling approaches for creating hetero-oligomers are identical to those for creating homo-oligomers [17]. In brief, the hetero-dimer contains one tau monomer and one amylin monomer, while the hetero-tetramer contains two tau and two amylin monomers. The size of the simulation box was ~21 × 11 × 11 nm^3^ for the hetero-dimer and ~21 × 21 × 11 nm^3^ for the hetero-tetramer. Each CG simulation was performed under the physiological conditions of 1 atmospheric pressure, 310 K, and 0.1 M NaCl using the Martini-2.20 CG force field [20], and it was run on the GROMACS-4.67 MD simulation program [37]. To reveal the distribution of the hydrophobic residues in each protein, a hydropathy index (hydrophobicity) vs. residue number plot, or hydrophobicity profile, was determined for each tau or amylin monomer based on its primary sequence. Briefly, the hydropathic character of each amino acid in the protein was plotted as a function of the residue number, and a 5-point moving average was further applied to create the continuous hydrophobicity profile of a given protein [38,39]. Detailed discussions of the generation of the hydrophobic profiles of tau and amylin are further given elsewhere [13,14]. 

### 4.2. Modeling Asymmetric Raft Membranes

Our asymmetric raft membrane, i.e., PS-raft or GM-raft, is a lipid bilayer containing saturated dipalmitoyl-PC, unsaturated linoleoyl-PC (DLPC), cholesterol (CHOL), and 1-palmitoyl-2-oleoyl-PS (POPS) or monosialotetrahexosylganglioside (GM1) in water with a size of ~22 × 22 × 20 nm^3^, respectively. Descriptions of the modeling of these raft membranes are given elsewhere [13]. Briefly, the PS-raft contains 162 POPS, 666 DPPC, 540 DLPC, 576 CHOL, and 65,365 water molecules, and the GM-raft contains 36 GM1, 709 DPPC, 407 DLPC, 410 CHOL, and 56,114 water molecules. Under the physiological conditions of atmospheric pressure, 310 K, and 0.1 M NaCl, the lipids are phase-separated into ordered DPPC-rich and CHOL-rich Lo domains, disordered DLPC-rich Ld domains, and mixed DPPC-DLPC or Lod domains for up to 15 μs in CG simulations [20,37]. In addition, PS- or GM1-clusters within the Lo domain on one lipid leaflet were maintained for the PS-raft or GM-raft, respectively, throughout the entire CG simulation [13,15]. Note that the PS-raft and GM-raft described in this work are identical to the raft membranes used in our homo-oligomeric models, i.e., homo-tau and homo-amylin oligomers of different sizes [13,15]. 

### 4.3. Multiscale Simulations of Hetero-Oligomer Binding to Asymmetric Raft Membranes

To simulate hetero-oligomer binding to either PS- or GM-raft membrane in CG resolution, each equilibrated hetero-oligomer in solution was placed at a distance above the raft membrane surface containing the PS- or GM-clusters without contacting any lipid atoms of the membrane. The designs of the initial hetero-oligomer/raft complexes are identical to those of the initial homo-oligomer/raft complexes in our previous studies [13,15]. Briefly, to improve phase sampling of protein-membrane binding events, three independent simulation replicates were created for each initial hetero-oligomer/raft complex: replicate 1, replicate 2, and replicate 3. Here, replicate 1 represents placing the protein above the center of the surface of the lipid leaflet (upper leaflet), with the minimum distance between any atom of the protein and any atom of the lipid > 5 nm. Replicates 2 and 3 were subsequently created with the protein position shifted +2 nm and -2 nm along the *x*-direction relative to the protein position of replicate 1, respectively. Upon generating the initial structure of the complex, the same CG MD simulation procedure for preparing the oligomer or raft membrane in solution was performed for 15 μs. The MD simulation visualization program VMD [40] was used to monitor the protein/lipid binding events for all the replicates. 

After the 15 μs-long CG simulation, the equilibrated hetero-oligomer/raft complex from each replicate containing the membrane-bound oligomer was converted to the AA structure by a CG-to-AA resolution transformation procedure [18]. However, instead of the Martini CG force fields, the atomistic AMBER99SB [41] for proteins and SLIPIDS [42,43] for lipids force fields were used in all AA MD simulations up to 100 ns. 

Note that identical CG and AA MD simulation protocols and data analysis procedures for homo-oligomer systems were directly applied to the current hetero-oligomer systems. 

### 4.4. Classifications of Lipid Domains and Annular Lipids

The phase-separated Lo, Ld, and Lod domains of the raft membranes in the absence and presence of membrane-bound oligomers were classified using a lipid clustering tool, *g_select*, from GROMACS [37]. This tool is based on a proximity threshold of 0.5 nm between any atoms of DPPC and DLPC, with details given elsewhere [44]. The selection of the 0.5 nm threshold is arbitrary and has been used as a common metric in our previous computational studies to define our annular lipid shell in different protein/membrane models [13,14,15,17,44,45]. Briefly, in the presence or absence (control) of membrane-bound hetero-oligomers, all PC molecules in the raft membrane were classified into Lo-DPPC, Ld-DPPC, and Lod-PC. Here, the Lo-DPPC group consists of DPPC in the Lo domain, the Ld-DLPC group consists of DLPC in the Ld domain, and the Lod-PC group contains both DPPC and DLPC in the boundary Lod domain. Similarly, all CHOL molecules were classified into Lo-CHOL, Ld-CHOL, and Lod-CHOL groups, for which at least one CHOL atom is within 0.5 nm of the PC lipid atoms in the Lo, Ld, and Lod domains, respectively. Therefore, the Lo-DPPC and Lo-CHOL groups consist of lipid molecules residing in the highly ordered Lo domains. The Ld-DLPC and Ld-CHOL groups represent the lipid molecules made up of the highly disordered Ld domains. Finally, the Lod-PC and Lod-CHOL represent the lipid molecules in the boundary region between the Lo and Ld domains. 

The same *g_select* tool was also used to classify the annular lipid (AL) shell from each oligomer-raft complex upon hetero-oligomer binding, with details given elsewhere [44]. Briefly, if an atom of any lipid is within 0.5 nm of an atom of an oligomer, that lipid is assigned to the AL shell. The non-annular lipid (nAL) region was also classified. This nAL region contains lipids that are outside the AL lipid shell. 

### 4.5. Characterizations of Membrane Binding Behaviors of Hetero-Oligomers

The lipid-binding kinetics and residue-resolved binding patterns were analyzed using an analysis tool, *mindist*, from GROMACS [37], and the details are given [44]. Briefly, the time-dependent protein-lipid or protein-water minimum distance, defined as the minimum distance between any protein atom and the atom of its binding lipid or water neighbors (*mindist*), was recorded during the entire CG or AA MD simulation. In addition, the number of atom contacts of the mindist within an interaction threshold (2 nm) vs. simulation time was also determined. Finally, the time-averaged mindist vs. protein residue number over the last 5 μs of the CG simulation or the last 50 ns of the AA simulation was calculated. Collectively, these three parameters, mindist vs. time (upper panel), number of contacts vs. time (mid panel), and mindist vs. protein-residue number (bottom panel), were presented as a 3-panel plot of each replicate. Finally, the binding energetics, i.e., the nonbonded potential energies between the oligomer atoms and lipid (or water) atoms, were collected using the energy calculation tool, *energy*, from GROMACS [37] for each simulation time frame. Both Lennard-Jones and Coulomb energies were determined with an energy sampling threshold of ~1.2 nm. The details of these energy calculations are given elsewhere [44].

### 4.6. Characterizations of Lipid Orientational Order of Raft Membranes 

To assess protein-induced membrane disruption, the lipid orientational order parameter vs. acyl chain carbon number, or lipid order profile, in the AL shell and nAL region was calculated using the tool *order* from GROMACS [37]. This lipid order profile measures the tilt of three sequentially connected carbon atoms along the PC acyl chains with respect to the normal of the bilayer and provides a transverse (along the bilayer normal) profile of the lipid acyl chain ordering in the AL shell and the nAL region. Details of the lipid order profile calculation are given elsewhere [44].

### 4.7. Secondary Structures of Membrane-Bound Hetero-Oligomers

The residue-resolved secondary structure of the membrane-bound oligomer was calculated from the AA simulation using the tool *do_dssp* from GROMACS [37]. This tool is based on the Define Secondary Structure of Proteins (DSSP) [46] algorithm. To streamline the analysis, we grouped the beta-sheet and beta-bridge structures into a single “beta” group, the three helical structures, alpha helix (A-helix), p-helix (or 5-helix), and 310 helix (3-helix), into a single “alpha” group, and the bend and coil into a single “random” group at each AA simulation time frame. 

### 4.8. Protein-Residue Contact Maps of Hetero-Oligomers

To evaluate the interactions between residues from different types of proteins within the hetero-oligomer, 3D protein residue-contact maps describing the color-coded residue-residue minimum distances among all participating protein residues along the *x*- and *y*-axes were calculated using the tool *g_mdmat* from GROMACS [37] and a statistical and molecular interaction analysis tool, CONAN [47]. Time-averaged protein residue-contact maps with a standard deviation were generated. Details of residue-contact map generation were described in our previous studies [14,44].

## 5. Conclusions

In conclusion, this study indicates that the hetero-oligomers bind strongly to the anionic PS- and GM-clusters in the raft-like lipid domains that mimic the inner and outer leaflets of the neuronal plasma membrane, respectively. The protein-lipid interactions are mainly hydrophobic and hydrophilic, involving both amylin and tau peptides in PS-raft and GM-raft, respectively. We observed that the protein-lipid binding affinity follows the ranking order of GM1 > POPS > DPPC ≈ DLPC > CHOL, indicating the importance of GM1- and PS-clusters in protein binding. The membrane-disruptive effects of hetero-oligomers depend on the oligomer size, lipid type, and raft type. Overall, the hetero-tetramer perturbed the DPPC and POPS lipids in the PS-raft but only the GM1 in the GM-raft as effectively as the homo-tau-tetramer. Like homo-oligomers, all hetero-oligomers express beta and alpha-helical structures on both PS- and GM-rafts, supporting the surface-induced protein effects on raft membranes. We discovered that the presence of amylin-tau interaction within the hetero-dimer strongly promotes alpha helix formation when compared with amylin or tau alone for both raft membranes. This synergetic effect of amylin-tau provides new molecular insight into the cross-talk between diabetics and Alzheimer’s. Finally, our new in silico structures of membrane-bound hetero-tau-amylin oligomers are useful for future therapeutic interventions targeting both intracellular and extracellular membrane tau-amylin oligomers in the early pathogenesis of Alzheimer’s in the presence of both toxic tau and amylin aggregates in the brain. 

## Data Availability

The data presented in this study are available in article and Supplementary Materials.

## Supplementary Materials

The following supporting information can be downloaded at: https://www.mdpi.com/article/10.3390/molecules29030740/s1. Figure S1: Modeling of homogeneous amylin and tau oligomers, Figure S2: Modeling heterogeneous tau-amylin oligomers in solution, Figure S3: Minimum distance analysis of 1tam binding to the PS-raft membrane, Figure S4: Minimum distance analysis of 2tam binding to the PS-raft membrane, Figure S5: Minimum distance analysis of 1tam binding to the GM-raft membrane, Figure S6: Minimum distance analysis of 2tam binding to the GM-raft membrane, Figure S7: Annular lipid compositions of homo- and hetero-oligomers in the PS-raft, Figure S8: Annular lipid compositions of homo- and hetero-oligomers in the GM-raft, Figure S9: Protein structures of membrane-bound hetero-oligomers, Figure S10: Effects of amylin on protein folding of tau in hetero-oligomers, Figure S11: Effects of tau on protein folding of amylin in hetero-oligomers, Figure S12: Contact maps of hetero-oligomers in solution, Figure S13: Contact maps of hetero-oligomers in the CO-raft, Figure S14: Contact maps of hetero-oligomers in the PS-raft, and Figure S15: Contact maps of hetero-oligomers in the GMS-raft; Table S1: Summary of ANOVA Analysis of protein effect on alpha helix formation; Video S1: Creation of 1tam in solution (0–5 μs; 0.1 μs/frame), Video S2: Creation of 2tam in solution (0–5 μs; 0.1 μs/frame), Video S3: 1tam binding to CO-raft. (0–15 μs; 0.1 μs/frame; Lateral view), Video S4: 1tam binding to CO-raft. (0–15 μs; 0.1 μs/frame; Transverse view), Video S5: 1tam binding to PS-raft. (0–15 μs; 0.1 μs/frame; Lateral view), Video S6: 1tam binding to PS-raft. (0–15 μs; 0.1 μs/frame; Transverse view), Video S7: 1tam binding to GM-raft (0–15 μs; 0.1 μs/frame; Lateral view), and Video S8: 1tam binding to GM-raft (0–15 μs; 0.1 μs/frame; Transverse view). All the videos can be found at https://data.mendeley.com/datasets/8vb23c48ph/2 (accessed on 20 December 2023).

## Abbreviations

AA	all-atom
CG	coarse-grained
MD	molecular dynamics
1am	monomeric amylin
2am	dimeric amylin homo-amylin oligomer
4am	tetrameric amylin in homo-amylin oligomer
1tau	monomeric tau
2tau	dimeric tau in homo-tau oligomer
4tau	tetrameric tau in homo-tau oligomer
1tam	dimeric tau-amylin oligomer
2tam	tetrameric tau-amylin oligomer
1am’	monomeric amylin in 1tam
2am’	dimeric amylin in 2tam
1tau’	monomeric tau in 1tam
2tau’	dimeric tau in 2tam
PC	phosphatidylcholine
PS	phosphatidylserine
CHOL	cholesterol
DPPC	dipalmitoyl-PC
DLPC	dilinoleoyl-PC
POPS	1-palmitoyl, 2-oleoyl-PS
GM1	monosialotetrahexosylganglioside
Lo	liquid-ordered
Ld	liquid-disordered
Lod	mixed Lo/Ld
AL	annular lipid
nAL	non-annular lipid
*mindist*	minimum distance
DSSP	Define Secondary Structure of Proteins

## References

[1] Gerson J.E., Castillo-Carranza D.L., Kayed R. (2014). Advances in therapeutics for neurodegenerative tauopathies: Moving toward the specific targeting of the most toxic tau species. ACS Chem. Neurosci..

[2] Khan A.N., Khan R.H. (2022). Protein misfolding and related human diseases: A comprehensive review of toxicity, proteins involved, and current therapeutic strategies. Int. J. Biol. Macromol..

[3] Milardi D., Gazit E., Radford S.E., Xu Y., Gallardo R.U., Caflisch A., Westermark G.T., Westermark P., Rosa C., Ramamoorthy A. (2021). Proteostasis of Islet Amyloid Polypeptide: A Molecular Perspective of Risk Factors and Protective Strategies for Type II Diabetes. Chem. Rev..

[4] Bortoletto A.S., Parchem R.J. (2023). A pancreatic player in dementia: Pathological role for islet amyloid polypeptide accumulation in the brain. Neural Regen. Res..

[5] Zhang G., Meng L., Wang Z., Peng Q., Chen G., Xiong J., Zhang Z. (2022). Islet amyloid polypeptide cross-seeds tau and drives the neurofibrillary pathology in Alzheimer’s disease. Mol. Neurodegener..

[6] Zhu H., Tao Q., Ang T.F.A., Massaro J., Gan Q., Salim S., Zhu R.Y., Kolachalama V.B., Zhang X., Devine S. (2019). Association of Plasma Amylin Concentration With Alzheimer Disease and Brain Structure in Older Adults. JAMA Netw. Open.

[7] Wijesekara N., Goncalves R.A., Ahrens R., Ha K., De Felice F.G., Fraser P.E. (2021). Combination of human tau and islet amyloid polypeptide exacerbates metabolic dysfunction in transgenic mice. J. Pathol..

[8] Arya S., Claud S.L., Cantrell K.L., Bowers M.T. (2019). Catalytic Prion-Like Cross-Talk between a Key Alzheimer’s Disease Tau-Fragment R3 and the Type 2 Diabetes Peptide IAPP. ACS Chem. Neurosci..

[9] Cebecauer M., Amaro M., Jurkiewicz P., Sarmento M.J., Sachl R., Cwiklik L., Hof M. (2018). Membrane Lipid Nanodomains. Chem. Rev..

[10] Ingolfsson H.I., Bhatia H., Zeppelin T., Bennett W.F.D., Carpenter K.A., Hsu P.C., Dharuman G., Bremer P.T., Schiott B., Lightstone F.C. (2020). Capturing Biologically Complex Tissue-Specific Membranes at Different Levels of Compositional Complexity. J. Phys. Chem. B.

[11] Chiricozzi E., Lunghi G., Di Biase E., Fazzari M., Sonnino S., Mauri L. (2020). GM1 Ganglioside Is A Key Factor in Maintaining the Mammalian Neuronal Functions Avoiding Neurodegeneration. Int. J. Mol. Sci..

[12] Kim H.Y., Huang B.X., Spector A.A. (2014). Phosphatidylserine in the brain: Metabolism and function. Prog. Lipid Res..

[13] Cheng K.H., Graf A., Lewis A., Pham T., Acharya A. (2022). Exploring Membrane Binding Targets of Disordered Human Tau Aggregates on Lipid Rafts Using Multiscale Molecular Dynamics Simulations. Membranes.

[14] Lewis A., Pham T., Nguyen N., Graf A., Cheng K.H. (2023). Lipid domain boundary triggers membrane damage and protein folding of human islet amyloid polypeptide in the early pathogenesis of amyloid diseases. Biophys. Chem..

[15] Nguyen N., Lewis A., Pham T., Sikazwe D., Cheng K.H. (2023). Exploring the Role of Anionic Lipid Nanodomains in the Membrane Disruption and Protein Folding of Human Islet Amyloid Polypeptide Oligomers on Lipid Membrane Surfaces Using Multiscale Molecular Dynamics Simulations. Molecules.

[16] Nguyen P.H., Ramamoorthy A., Sahoo B.R., Zheng J., Faller P., Straub J.E., Dominguez L., Shea J.E., Dokholyan N.V., De Simone A. (2021). Amyloid Oligomers: A Joint Experimental/Computational Perspective on Alzheimer’s Disease, Parkinson’s Disease, Type II Diabetes, and Amyotrophic Lateral Sclerosis. Chem. Rev..

[17] Santos N., Segura L., Lewis A., Pham T., Cheng K.H. (2023). Molecular Mechanisms of Protein-Lipid Interactions and Protein Folding of Heterogeneous Amylin and Tau Oligomers on Lipid Nanodomains That Link to Alzheimer’s. Macromol.

[18] Wassenaar T.A., Pluhackova K., Bockmann R.A., Marrink S.J., Tieleman D.P. (2014). Going Backward: A Flexible Geometric Approach to Reverse Transformation from Coarse Grained to Atomistic Models. J. Chem. Theory Comput..

[19] Ashraf G.M., Greig N.H., Khan T.A., Hassan I., Tabrez S., Shakil S., Sheikh I.A., Zaidi S.K., Akram M., Jabir N.R. (2014). Protein misfolding and aggregation in Alzheimer’s disease and type 2 diabetes mellitus. CNS Neurol. Disord. Drug Targets.

[20] Monticelli L., Kandasamy S.K., Periole X., Larson R.G., Tieleman D.P., Marrink S.J. (2008). The MARTINI Coarse-Grained Force Field: Extension to Proteins. J. Chem. Theory Comput..

[21] Brender J.R., Hartman K., Reid K.R., Kennedy R.T., Ramamoorthy A. (2008). A single mutation in the nonamyloidogenic region of islet amyloid polypeptide greatly reduces toxicity. Biochemistry.

[22] Brender J.R., McHenry A.J., Ramamoorthy A. (2012). Does cholesterol play a role in the bacterial selectivity of antimicrobial peptides?. Front. Immunol..

[23] Hasan M., Moghal M.M.R., Saha S.K., Yamazaki M. (2019). The role of membrane tension in the action of antimicrobial peptides and cell-penetrating peptides in biomembranes. Biophys. Rev..

[24] Lee E.Y., Srinivasan Y., de Anda J., Nicastro L.K., Tukel C., Wong G.C.L. (2020). Functional Reciprocity of Amyloids and Antimicrobial Peptides: Rethinking the Role of Supramolecular Assembly in Host Defense, Immune Activation, and Inflammation. Front. Immunol..

[25] Pinigin K.V., Kondrashov O.V., Jimenez-Munguia I., Alexandrova V.V., Batishchev O.V., Galimzyanov T.R., Akimov S.A. (2020). Elastic deformations mediate interaction of the raft boundary with membrane inclusions leading to their effective lateral sorting. Sci. Rep..

[26] Yang S.T., Kiessling V., Tamm L.K. (2016). Line tension at lipid phase boundaries as driving force for HIV fusion peptide-mediated fusion. Nat. Commun..

[27] Akimov S.A., Kuzmin P.I., Zimmerberg J., Cohen F.S. (2007). Lateral tension increases the line tension between two domains in a lipid bilayer membrane. Phys. Rev. E Stat. Nonlin Soft Matter Phys..

[28] Belicka M., Weitzer A., Pabst G. (2017). High-resolution structure of coexisting nanoscopic and microscopic lipid domains. Soft Matter.

[29] Risselada H.J., Marrink S.J. (2008). The molecular face of lipid rafts in model membranes. Proc. Natl. Acad. Sci. USA.

[30] de Wit G., Danial J.S., Kukura P., Wallace M.I. (2015). Dynamic label-free imaging of lipid nanodomains. Proc. Natl. Acad. Sci. USA.

[31] Sezgin E., Levental I., Mayor S., Eggeling C. (2017). The mystery of membrane organization: Composition, regulation and roles of lipid rafts. Nat. Rev. Mol. Cell Biol..

[32] Simons K., Sampaio J.L. (2011). Membrane organization and lipid rafts. Cold Spring Harb. Perspect. Biol..

[33] Li X., Lao Z., Zou Y., Dong X., Li L., Wei G. (2021). Mechanistic Insights into the Co-Aggregation of Aβ and hIAPP: An All-Atom Molecular Dynamic Study. J. Phys. Chem. B.

[34] Zhang M., Hu R., Ren B., Chen H., Jiang B., Ma J., Zheng J. (2017). Molecular Understanding of Abeta-hIAPP Cross-Seeding Assemblies on Lipid Membranes. ACS Chem. Neurosci..

[35] Brender J.R., Salamekh S., Ramamoorthy A. (2012). Membrane disruption and early events in the aggregation of the diabetes related peptide IAPP from a molecular perspective. Acc. Chem. Res..

[36] Sepehri A., Nepal B., Lazaridis T. (2021). Distinct Modes of Action of IAPP Oligomers on Membranes. J. Chem. Inf. Model..

[37] Hess B., Kutzner C., van der Spoel D., Lindahl E. (2008). GROMACS 4: Algorithms for Highly Efficient, Load-Balanced, and Scalable Molecular Simulation. J. Chem. Theory Comput..

[38] Han B., Tashjian A.H. (1998). User-friendly and versatile software for analysis of protein hydrophobicity. BioTechniques.

[39] Kyte J., Doolittle R.F. (1982). A simple method for displaying the hydropathic character of a protein. J. Mol. Biol..

[40] Humphrey W., Dalke A., Schulten K. (1996). VMD: Visual molecular dynamics. J. Mol. Graph..

[41] Maier J.A., Martinez C., Kasavajhala K., Wickstrom L., Hauser K.E., Simmerling C. (2015). ff14SB: Improving the Accuracy of Protein Side Chain and Backbone Parameters from ff99SB. J. Chem. Theory Comput..

[42] Blumer M., Harris S., Li M., Martinez L., Untereiner M., Saeta P.N., Carpenter T.S., Ingolfsson H.I., Bennett W.F.D. (2020). Simulations of Asymmetric Membranes Illustrate Cooperative Leaflet Coupling and Lipid Adaptability. Front. Cell Dev. Biol..

[43] Grote F., Lyubartsev A.P. (2020). Optimization of Slipids Force Field Parameters Describing Headgroups of Phospholipids. J. Phys. Chem. B.

[44] Pham T., Cheng K.H. (2022). Exploring the binding kinetics and behaviors of self-aggregated beta-amyloid oligomers to phase-separated lipid rafts with or without ganglioside-clusters. Biophys. Chem..

[45] Cheng S.Y., Cao Y., Rouzbehani M., Cheng K.H. (2020). Coarse-grained MD simulations reveal beta-amyloid fibrils of various sizes bind to interfacial liquid-ordered and liquid-disordered regions in phase separated lipid rafts with diverse membrane-bound conformational states. Biophys. Chem..

[46] Kabsch W., Sander C. (1983). Dictionary of protein secondary structure: Pattern recognition of hydrogen-bonded and geometrical features. Biopolymers.

[47] Mercadante D., Grater F., Daday C. (2018). CONAN: A Tool to Decode Dynamical Information from Molecular Interaction Maps. Biophys. J..

