# Supplementary material for: Multiscale Modeling of Macromolecular Interactions between Tau-Amylin Oligomers and Asymmetric Lipid Nanodomains That Link Alzheimer’s and Diabetic Diseases
Source: Molecules. 2024 Feb 5;29(3):740. doi: 10.3390/molecules29030740 (PMC10856442; doi:10.3390/molecules29030740)
Supplement: Supplementary file 1 [file molecules-29-00740-s001.zip › molecules-2819583-supplementary.pdf]

Supplementary Materials

Multiscale Modeling of Macromolecular Interactions between Heterogeneous Tau-Amylin Oligomers and Asymmetric Lipid Nanodomains that Link Alzheimer's and Diabetics Diseases

Natalia Santos [1], Luthary Segura [1], Amber Lewis [1], Thuong Pham [2] and Kwan H. Cheng [1,2,*]

[1] Neuroscience Department., Trinity University. San Antonio, TX 78212, USA; nsantos@trinity.edu (N.S.); lsegura1@trinity.edu (L.S.); alewis2@trinity.edu (A.L.)
[2] Physics Department., Trinity University, San Antonio, TX 78212, USA; tpham3@trinity.edu (T.P.)
* Correspondence: kcheng1@trinity.edu; Tel.: (+1-210-999-8469)

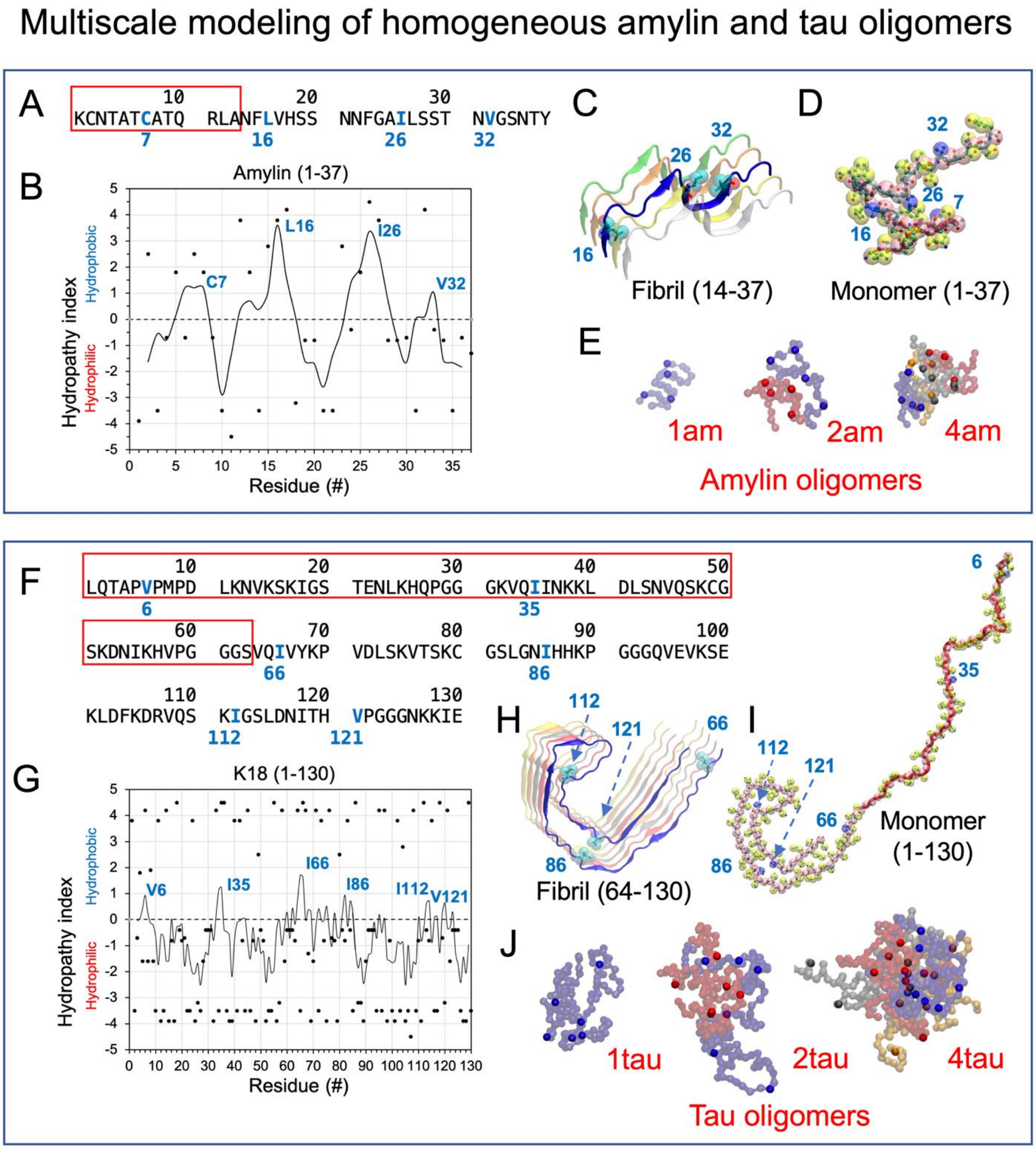

Figure S1. Modeling homogeneous amylin and tau oligomers. The primary sequences of amylin (**A**) and tau (**F**), and the corresponding hydrophobicity plots (**B**, **G**) are shown. The initial AA structure of a 37-residue-long amylin or a 130-residue-long tau monomer was created by extracting a chain from the CryoEM fibril structure (PDB: 6VW2 or 5O3L) and attaching a 13- or 63-residue-long coil to its *N*-terminus (**C**, **D**) or (**H**, **I**), respectively. After an AA-to-CG transformation followed by a 5-

μs-long-CG simulation, 1am (**E**) or 1tau (**J**) monomer in solution was created. By placing multiple monomers in different locations of a simulation box, amylin (**E**) or tau (**J**) oligomers were created via self-aggregation. All CG simulations were performed under physiological conditions of 0.1 M NaCl, 1 atmospheric pressure, and 310 K. The protein structures are shown in backbone ribbons with chain A in blue, chain B in red, chain C in gray, and chain D in orange. The major hydrophobic residues of amylin and tau as depicted by the hydrophobicity plots are shown in brighter beads. Detail modeling procedures of homogeneous amylin and tau oligomers have been published elsewhere. [1, 2].

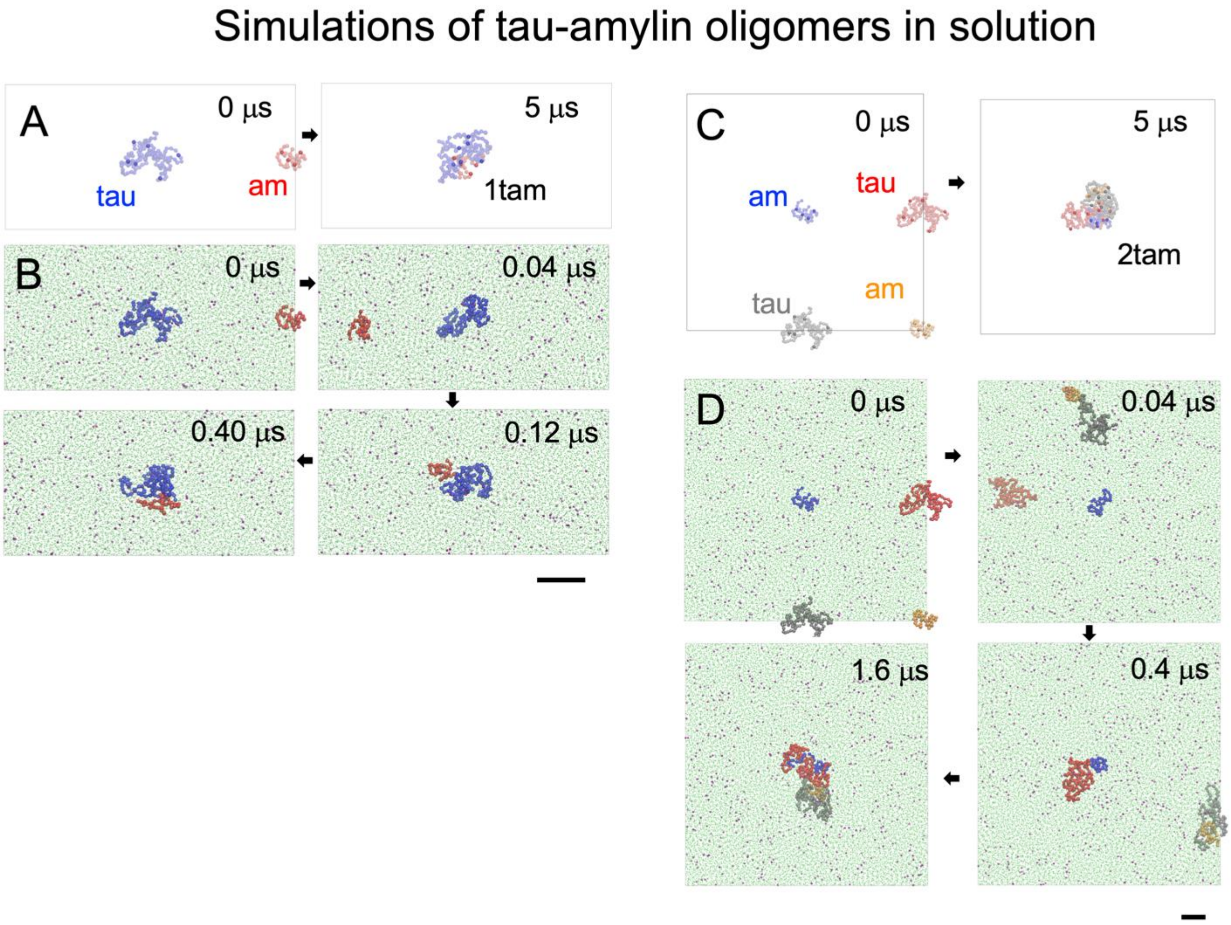

Figure S2. Modeling heterogeneous tau-amylin oligomers in solution. The initial (0 μs) and final (5 μs) CG structures of 1tam (**A**) and 2tam (**C**) in solution and their time evolutions of formation (**B**, **D**) from individual tau and am monomers are given. Water and ion CG atoms are in green dots and purple beads. The protein structures are shown in a backbone ribbon form with chain A in blue, chain B in red, chain C in gray, and chain D in orange. All CG simulations were performed in 0.1 M NaCl and under physiological conditions of 1 atmosphere and 310 K. A scale bar of 2 nm is shown. Detailed modeling procedures of heterogeneous tau-amylin oligomers have been published elsewhere. [3]

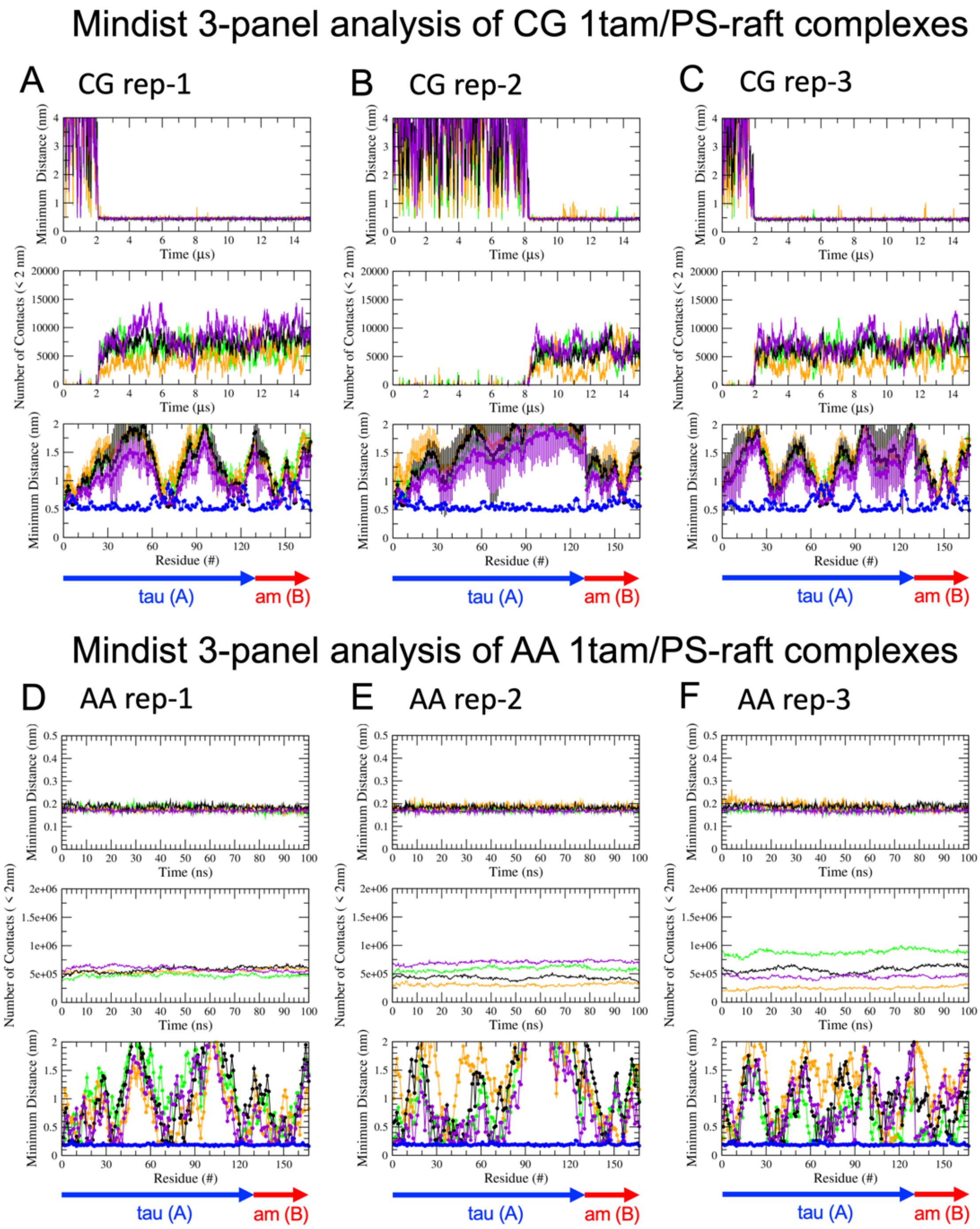

Figure S3. Minimum distance analysis of 1tam binding to the PS-raft membrane. Three-panel plots of the minimum distance (mindist) between protein and lipid (or water) atoms of three replicates, rep-1, rep-2, and rep-3, of the 1tam/raft complex in CG (**A**-**C**) and AA (**D**-**F**) simulations are shown. Each initial structure of the AA simulation was obtained from the 15 µs CG structure after a CG-to-AA transformation. The upper panel shows the mindist between protein and lipid atoms vs. time, the middle panel shows the number of contacts within 2 nm between protein and lipid atoms vs. time. The lower panel shows the time-averaged mindist between protein and lipid (or water) atoms vs. residue # over the last 5 µs for CG and the last 50 ns for AA. All mindist values are color-coded based on the lipid types, DPPC in green, DLPC in orange, CHOL in black, POPS in purple, and water in blue. The error bar represents the standard deviation of the mean. The protein residue locations of the tau and amylin (am) chains inside the 1tam are identified by the blue (chain A) and red (chain B) arrows, respectively.

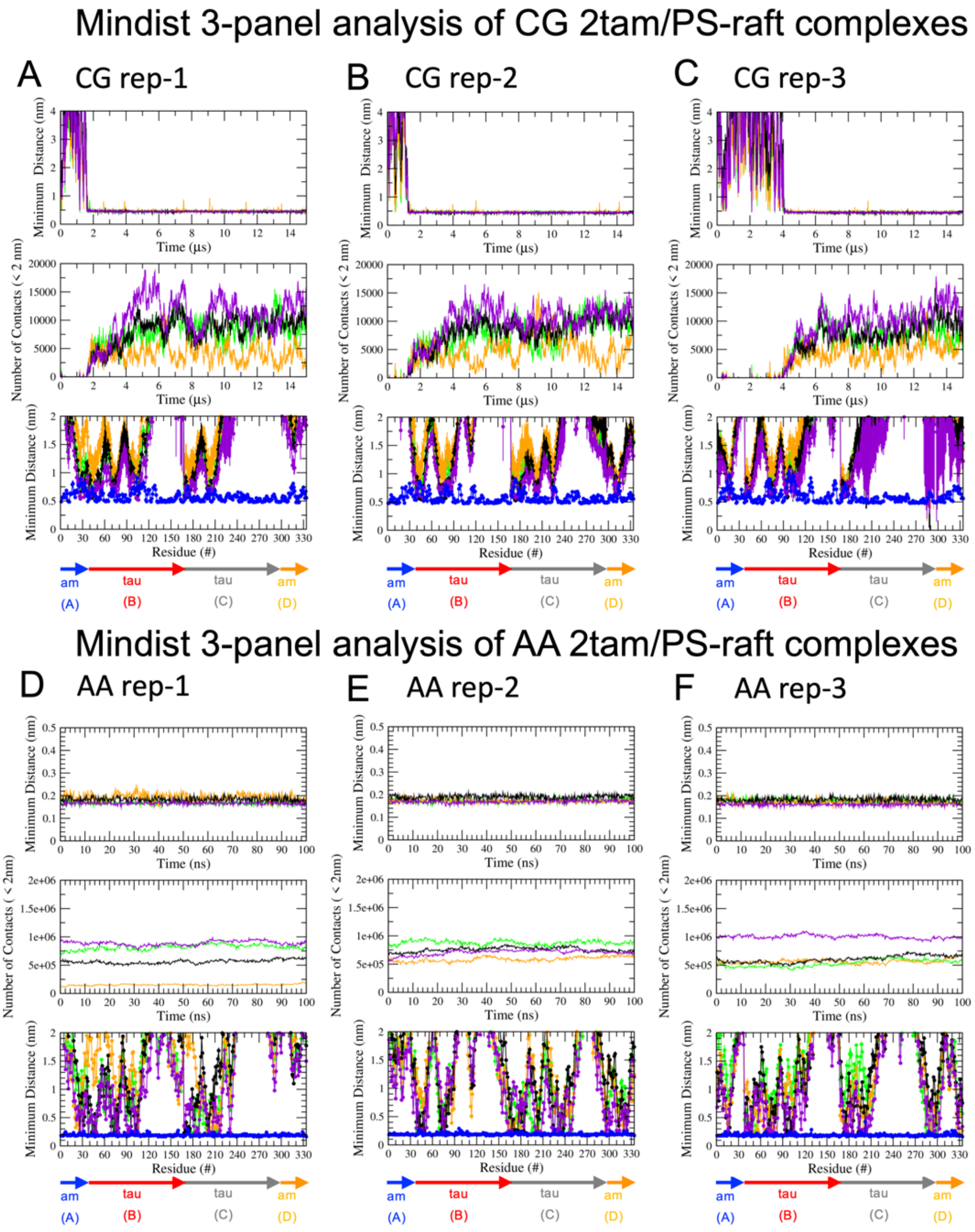

Figure S4. Minimum distance analysis of 2tam binding to the PS-raft membrane. Three-panel plots of the minimum distance (mindist) between protein and lipid (or water) atoms of three replicates, rep-1, rep-2, and rep-3, of the 2tam/raft complex in CG (**A**-**C**) and AA (**D**-**F**) simulations are shown. Each initial structure of the AA simulation was obtained from the 15 μs CG structure after a CG-to-AA transformation. The upper panel shows the mindist between protein and lipid atoms vs. time, the middle panel shows the number of contacts within 2 nm between protein and lipid atoms vs. time. The lower panel shows the time-averaged mindist between protein and lipid (or water) atoms vs. residue # over the last 5 μs for CG and the last 50 ns for AA. All mindist values are color-coded based on the lipid types, DPPC in green, DLPC in orange, CHOL in black, POPS in purple, and water in blue. The error bar represents the standard deviation of the mean. The protein residue locations of the tau and amylin (am) chains inside the 2tam are identified by the blue, red, gray, and orange arrows, corresponding to chains A (am), B (tau), C (tau), and D (am), respectively.

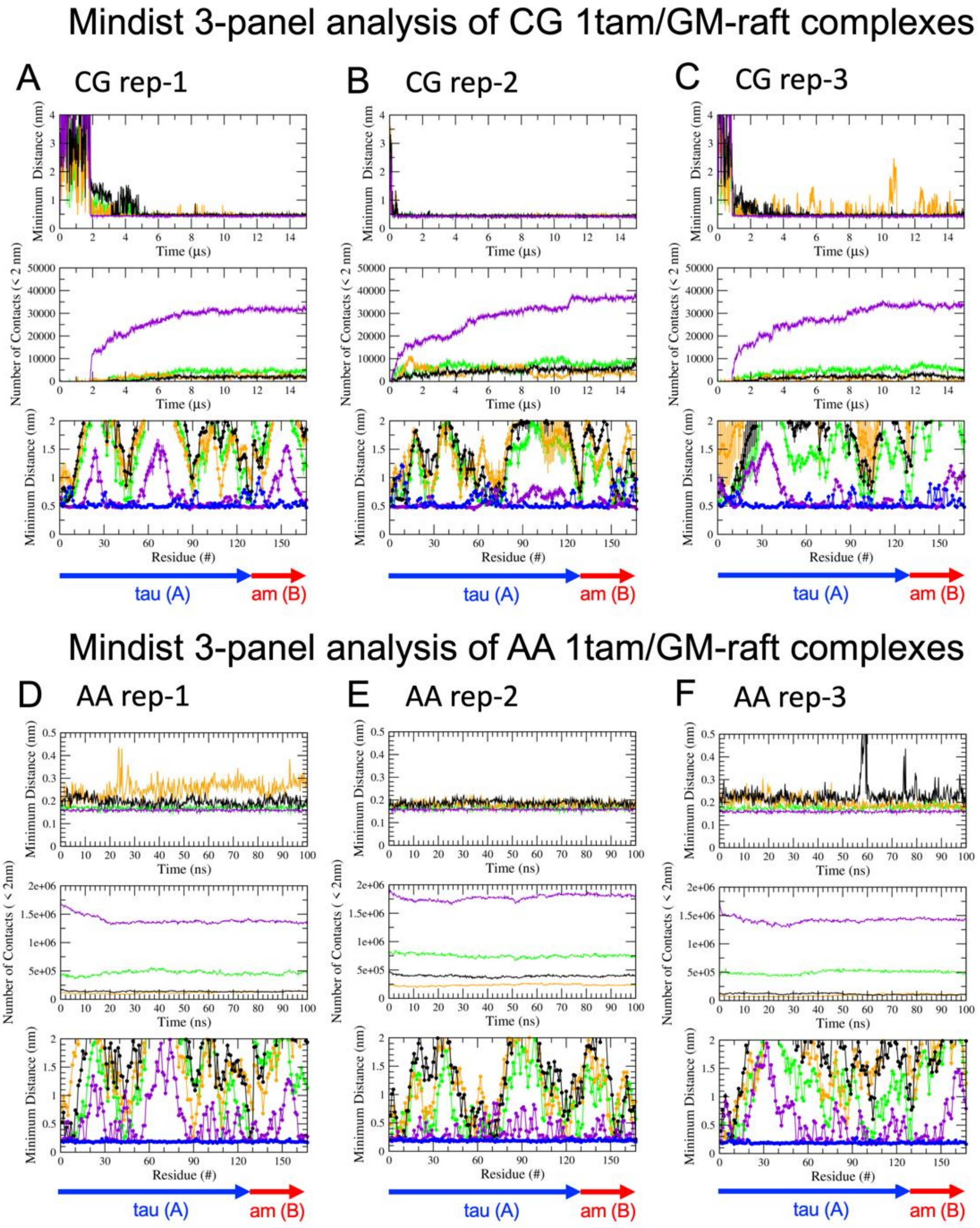

Figure S5. Minimum distance analysis of 1tam binding to the GM-raft membrane. Three-panel plots of the minimum distance (mindist) between protein and lipid (or water) atoms of three replicates, rep-1, rep-2, and rep-3, of the 1tam/raft complex in CG (**A**-**C**) and AA (**D**-**F**) simulations are shown. Each initial structure of the AA simulation was obtained from the 15 μs CG structure after a CG-to-AA transformation. The upper panel shows the mindist between protein and lipid atoms vs. time, the middle panel shows the number of contacts within 2 nm between protein and lipid atoms vs. time. The lower panel shows the time-averaged mindist between protein and lipid (or water) atoms vs. residue # over the last 5 μs for CG and the last 50 ns for AA. All mindist values are color-coded based on the lipid types, DPPC in green, DLPC in orange, CHOL in black, GM1 in purple, and water in blue. The error bar represents the standard deviation of the mean. The protein residue locations of the tau and amylin (am) chains inside the 1tam are identified by the blue (chain A) and red (chain B) arrows, respectively.

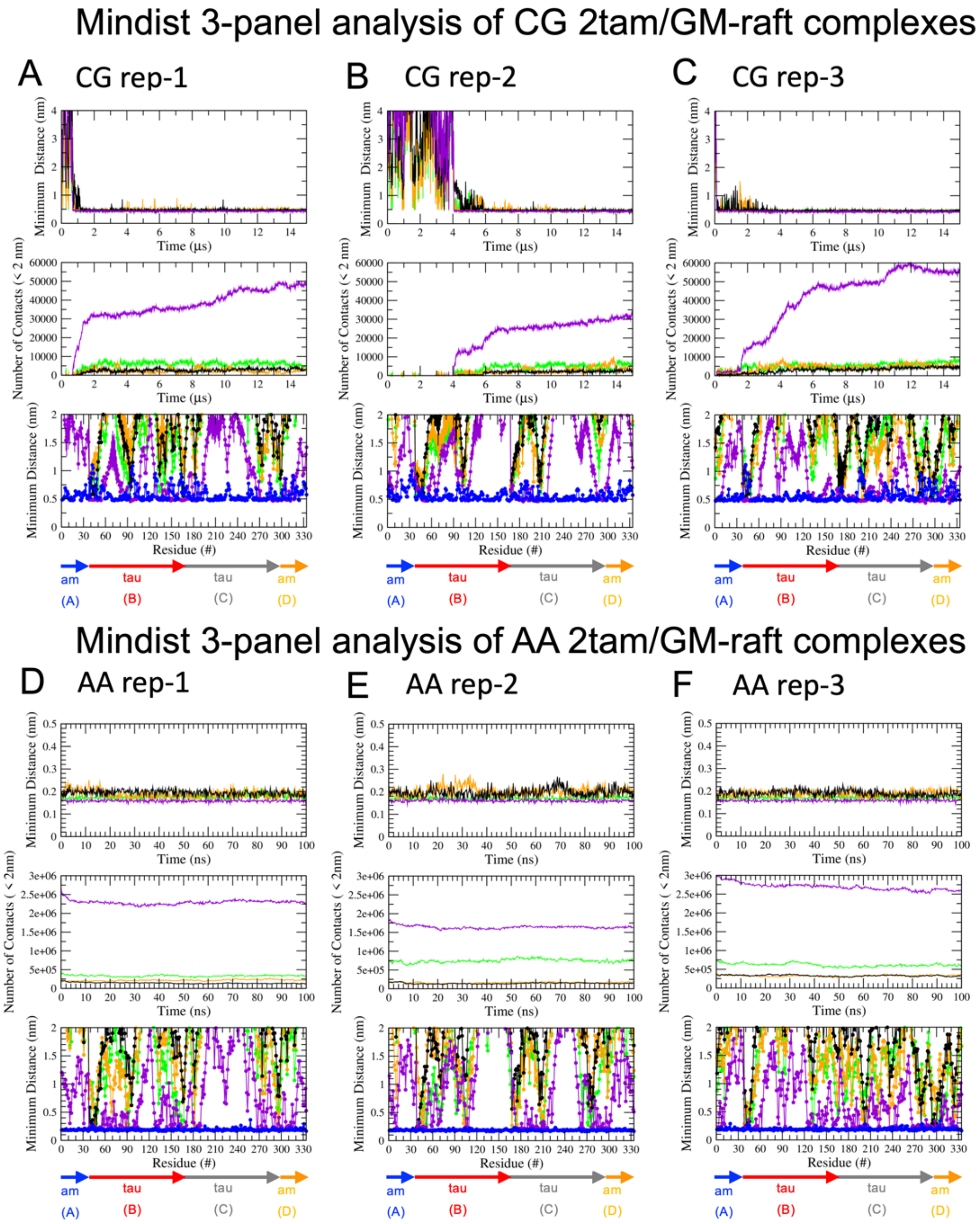

Figure S6. Minimum distance analysis of 2tam binding to the GM-raft membrane. Three-panel plots of the minimum distance (mindist) between protein and lipid (or water) atoms of three replicates, rep-1, rep-2, and rep-3, of the 2tam/raft complex in CG (**A**-**C**) and AA (**D**-**F**) simulations are shown. Each initial structure of the AA simulation was obtained from the 15 µs CG structure after a CG-to-AA transformation. The upper panel shows the mindist between protein and lipid atoms vs. time, the middle panel shows the number of contacts within 2 nm between protein and lipid atoms vs. time. The lower panel shows the time-averaged mindist between protein and lipid (or water) atoms vs. residue # over the last 5 µs for CG and the last 50 ns for AA. All mindist values are color-coded based on the lipid types, DPPC in green, DLPC in orange, CHOL in black, GM1 in purple, and water in blue. The error bar represents the standard deviation of the mean. The protein residue locations of the tau and amylin (am) chains inside the 2tam are identified by the blue, red, gray, and orange arrows, corresponding to chains A (am), B (tau), C (tau), and D (am), respectively.

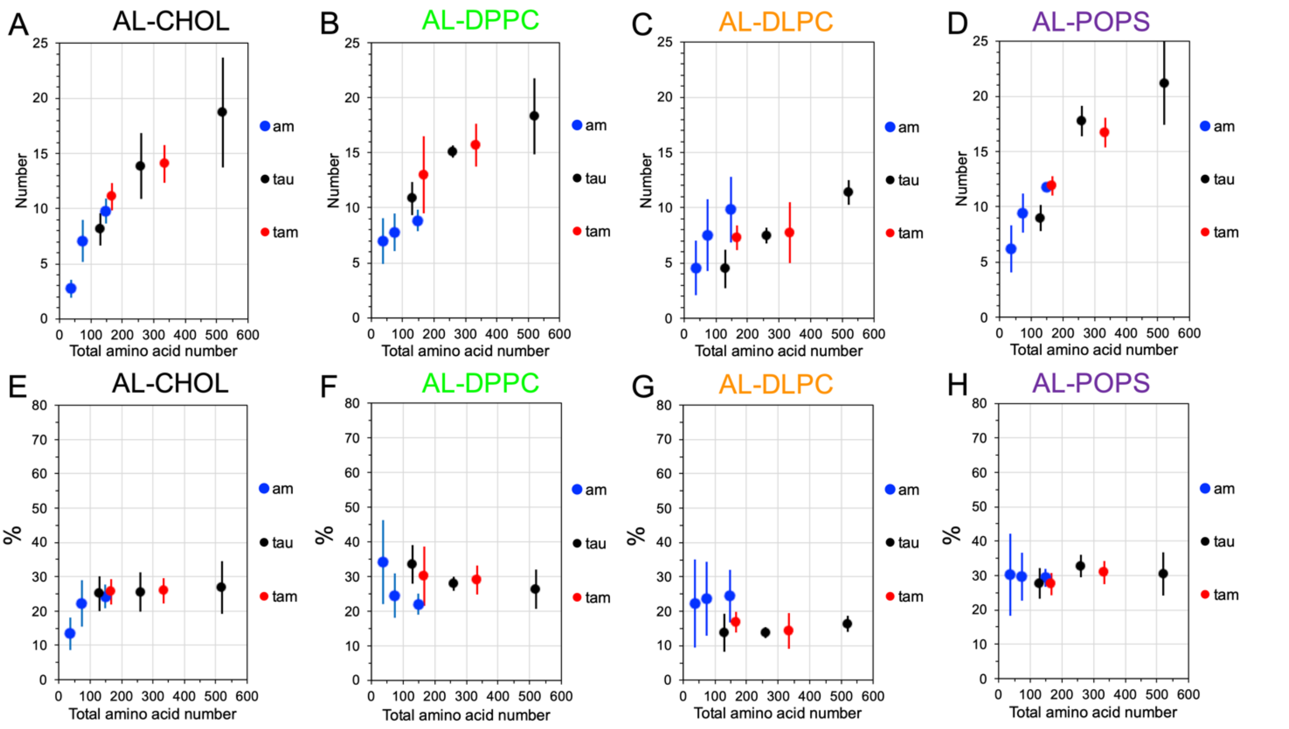

Figure S7. Annular lipid compositions of homo- and hetero-oligomers in the PS-raft. The total numbers (**A**-**D**) and percentages (**E**-**H**) of CHOL (**A**, **E**), DPPC (**B**, **F**), DLPC (**C**, **G**), and POPS (**D**, **H**) in the 0.5 nm annular lipid (AL) shells of homo-amylin (blue), homo-tau (black), and hetero-oligomers (red) are given. The oligomers are sorted according to their total amino acid numbers. Each data point represents the time- and replicate-averaged value over the last 50 ns of AA simulations and across all replicates. The error bars are standard errors of the means.

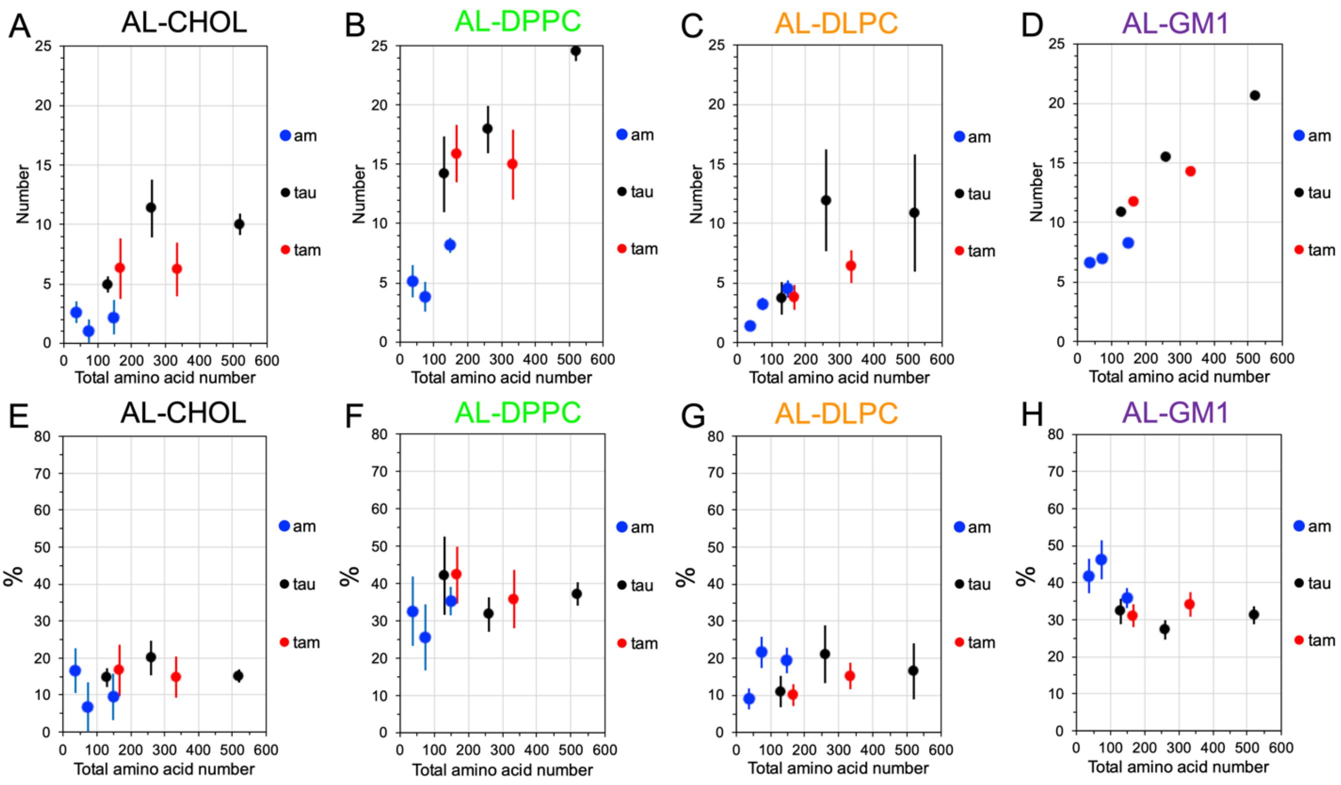

Figure S8. Annular lipid compositions of homo- and hetero-oligomers in the GM-raft. The total numbers (**A**-**D**) and percentages (**E**-**H**) of CHOL (**A**, **E**), DPPC (**B**, **F**), DLPC (**C**, **G**), and GM1 (**D**, **H**) in the 0.5 nm annular lipid (AL) shells of homo-amylin (blue), homo-tau (black), and hetero-oligomers (red) are given. The oligomers are sorted according to their total amino acid numbers. Each

data point represents the time- and replicate-averaged value over the last 50 ns of AA simulations and across all replicates. The error bars are standard errors of the means.

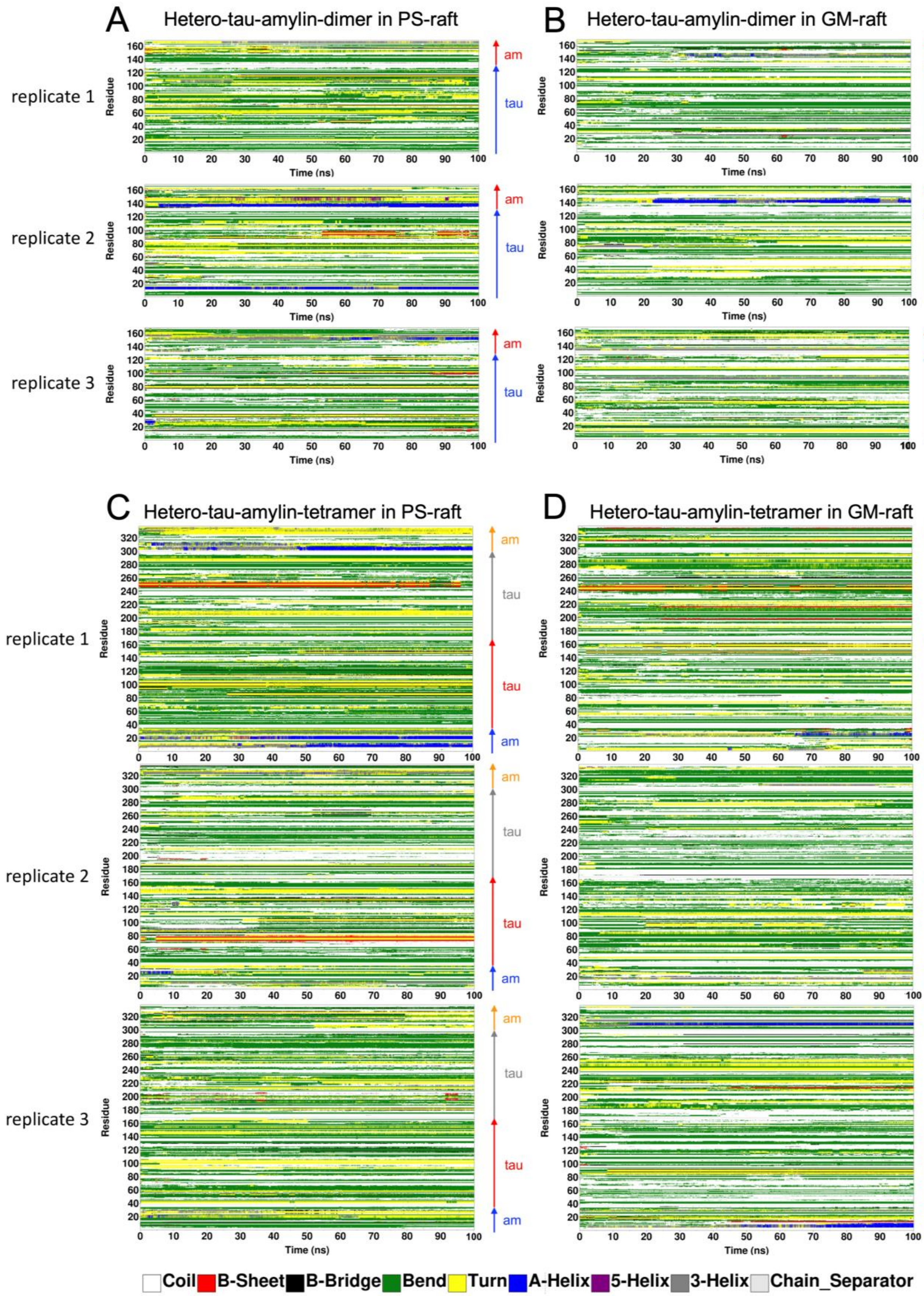

Figure S9. Protein structures of membrane-bound hetero-oligomers. The time (x-axis)- and residue (y-axis)-resolved secondary structures of the hetero-dimer in the PS-raft (**A**) and the GM-raft (**B**), as well as the hetero-tetramer in the PS-raft (**C**) and the GM-raft (**D**) in the DSSP format for all three

replicates are given. For the hetero-dimer, the residues belonging to the constituent tau and amylin (am) chains are identified by the blue and red arrows, respectively. For the tetramer, the residues belonging to the constituent tau and amylin chains are identified by the blue (am), red (tau), gray (tau), and orange (am) arrows.

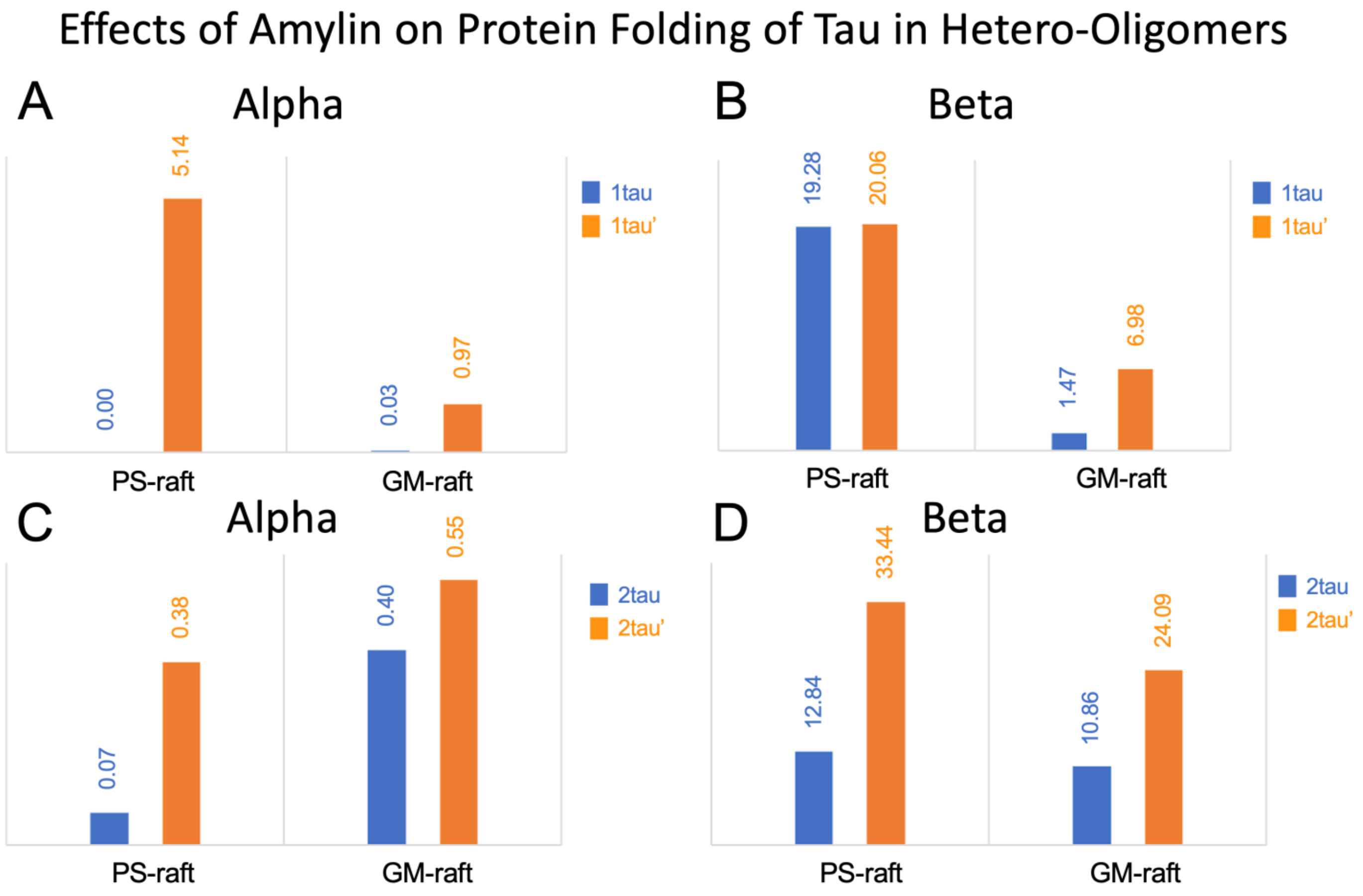

Figure S10. Effects of amylin on protein folding of tau in hetero-oligomers. The protein alpha (**A**, **C**) and beta (**B**, **D**) structures of the tau monomer in the hetero-dimer (1tau') and the tau-dimer in the hetero-tetramer (2tau') structures are compared alongside the tau monomer (1tau) and tau dimer (2tau) on the surfaces of PS-raft and GM-raft. Group difference analyses (see main text) were conducted for 1tau versus 1tau' as well as 2tau versus 2tau' with the matched total number of amino acid residues of 130 and 260, respectively. For 1tau versus 1tau' (**B**), there was a marginal significant effect of the surface with $p = 0.05$ on the beta structure formation, where the GM-raft induces greater beta structure than the PS-raft. All other relationships showed no significant differences, indicating that beta and alpha structure formation by tau does not differ significantly when initiating contact with amylin in the hetero-oligomers.

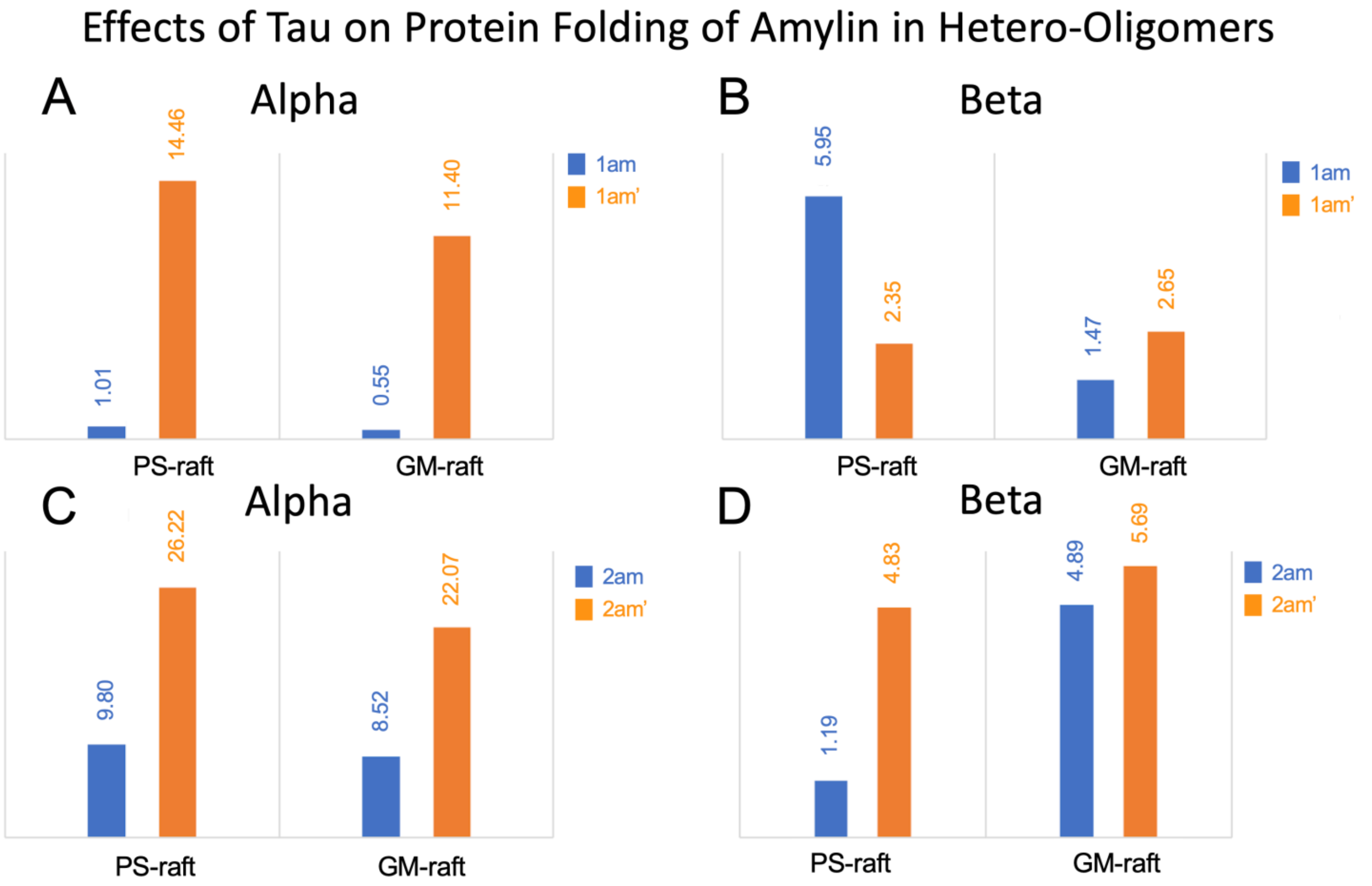

Figure S11. Effects of tau on protein folding of amylin in hetero-oligomers. The protein alpha (**A**, **C**) and beta (**B**, **D**) structures of amylin monomer in the hetero-dimer (1am') and the amylin-dimer in hetero-tetramer (2am') structures are compared alongside amylin monomer (1am) and amylin dimer (2am) on the surfaces of PS-raft and GM-raft. Group difference analyses (see main text) were conducted for 1am versus 1am′ as well as 2am versus 2am′ with the matched total number of amino acid residues of 37 and 74, respectively. There was a significant effect of alpha structure formation for 1 am vs. 1am′ (**A**) on both PS-raft and GM-raft with $p < 0.001$, but not for 2 am vs. 2am′ (**C**). There was no significant effect of the beta structure formation for either 1 am vs. 1am′ (**B**) or 2 am vs. 2am′ (**D**) across protein or raft membrane surface.

Hetero-tau-amylin-oligomers in solution

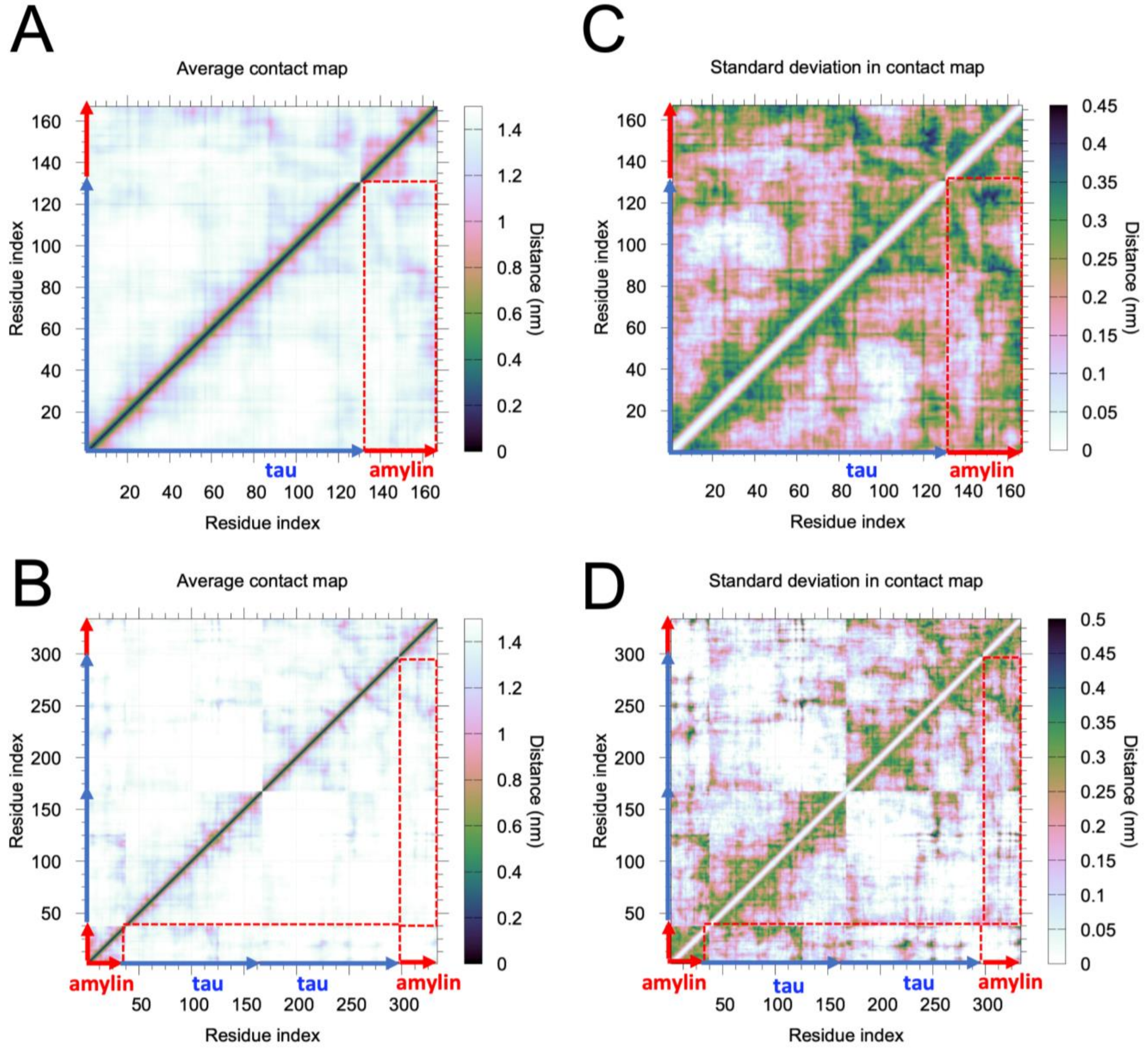

Figure S12. Contact maps of hetero-oligomers in solution. The average (**A**, **B**) and the standard deviation (**C**, **D**) of contact between each amino acid residue in a hetero-dimer (**A**, **C**) and a hetero-tetramer (**B**, **D**) in solution are shown. The contact regions between the residues of amylin (red arrow) and those of tau (blue arrow) are identified in dashed rectangles in the average contact maps. The color-coded data points represent the average and standard deviation of contact between each amino acid residue in the oligomer from the 5 μs of the CG simulation.

Hetero-tau-amylin-oligomers in CO-raft

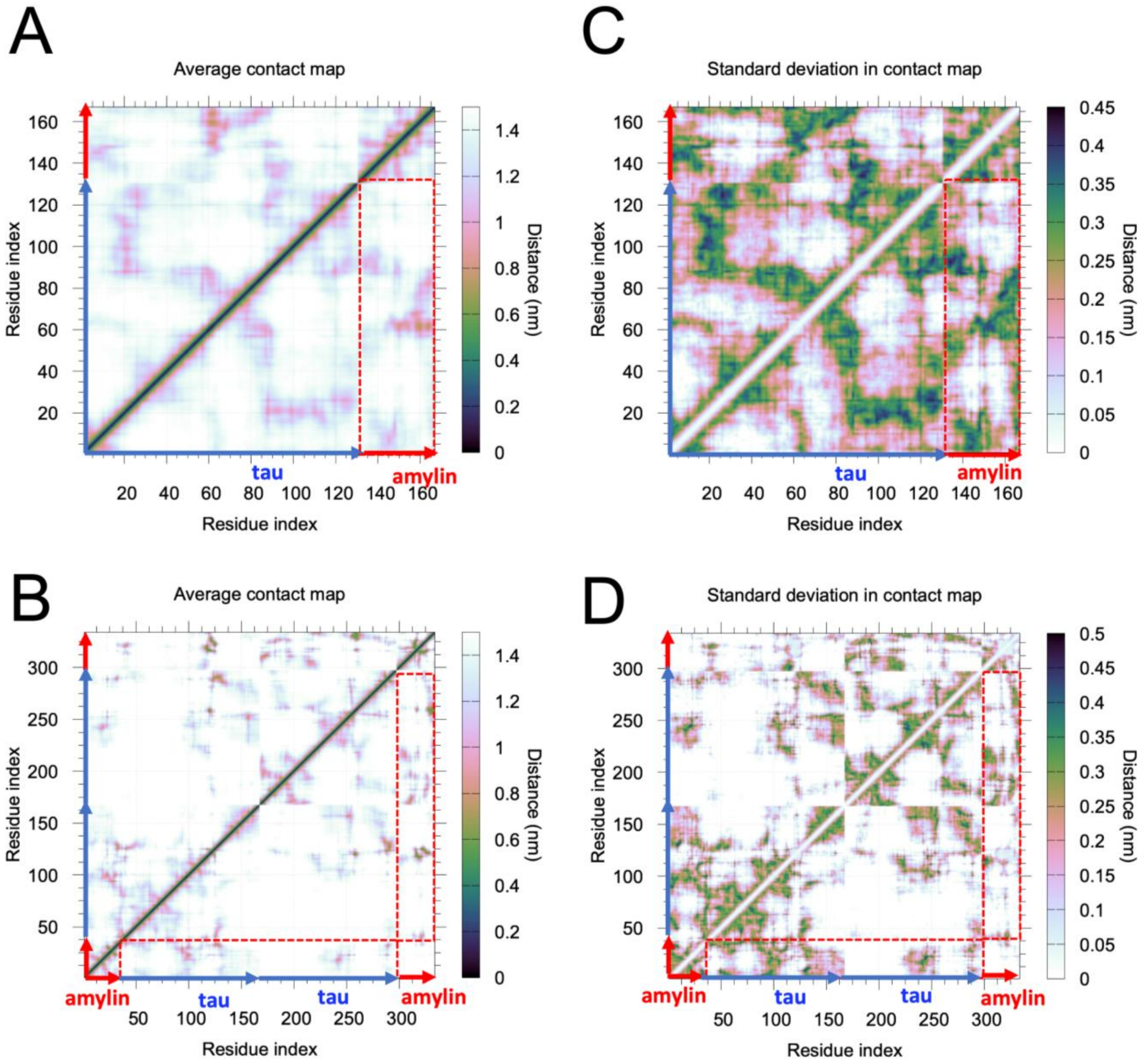

Figure S13. Contact maps of hetero-oligomers in the CO-raft. The average (**A**, **B**) and the standard deviation (**C**, **D**) of contact between each amino acid residue in a hetero-dimer (**A**, **C**) and a hetero-tetramer (**B**, **D**) on the raft membrane are shown. The contact regions between the residues of amylin (red arrow) and those of tau (blue arrow) are identified in dashed rectangles in the average contact maps. The color-coded data points represent the average and standard deviation of contact between each amino acid residue in the oligomer from the last 5 μs of the CG simulation.

Hetero-tau-amylin-oligomers in PS-raft

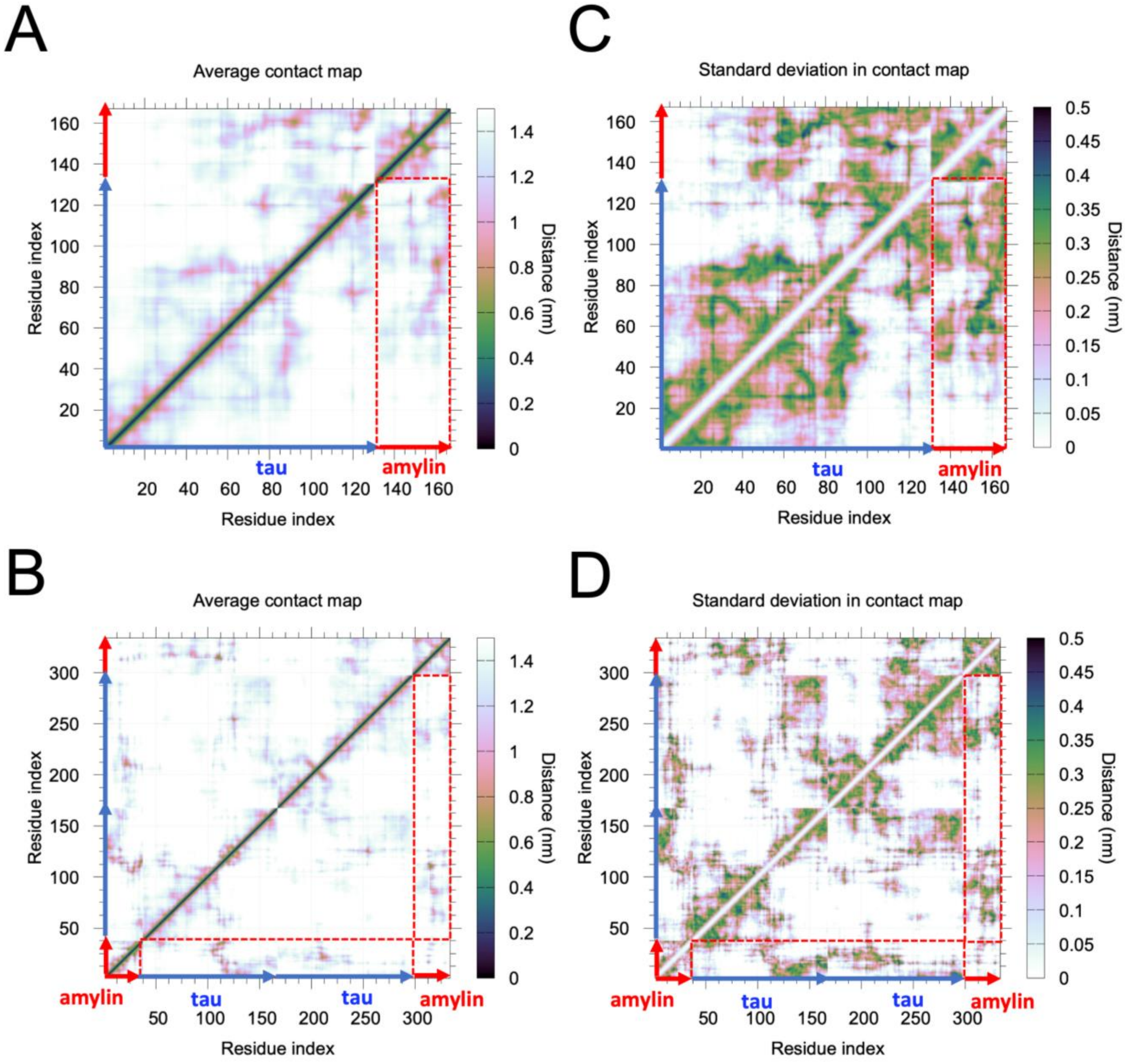

Figure S14. Contact maps of hetero-oligomers in the PS-raft. The average (**A**, **B**) and the standard deviation (**C**, **D**) of contact between each amino acid residue in a hetero-dimer (**A**, **C**) and a hetero-tetramer (**B**, **D**) on the raft membrane are shown. The contact regions between the residues of amylin (red arrow) and those of tau (blue arrow) are identified in dashed rectangles in the average contact maps. The color-coded data points represent the average and standard deviation of contact between each amino acid residue in the oligomer from the last 5 μs of the CG simulation.

Hetero-tau-amylin-oligomers in GM-raft

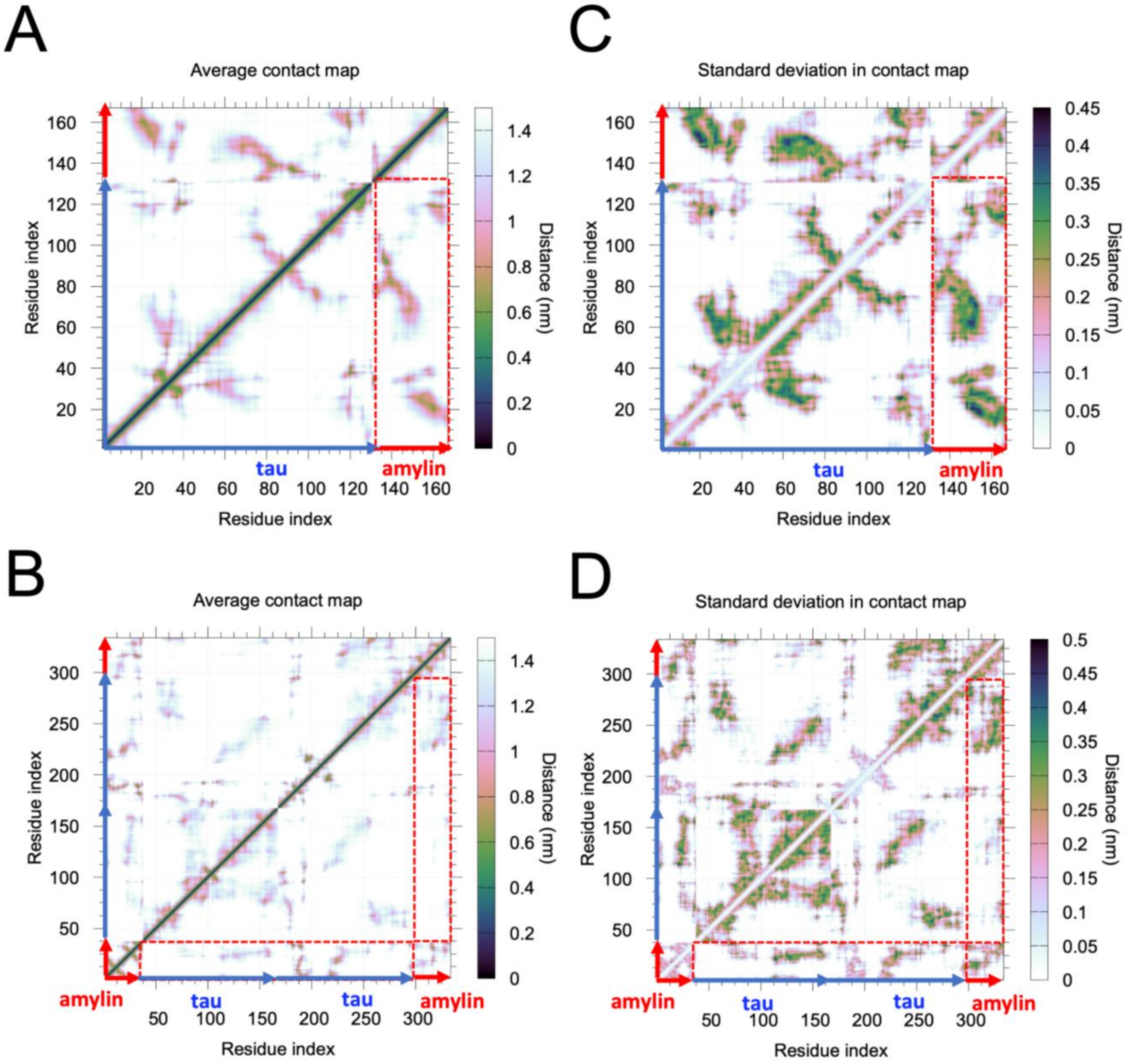

Figure S15. Contact maps of hetero-oligomers in the GM-raft. The average (**A**, **B**) and the standard deviation (**C**, **D**) of contact between each amino acid residue in a hetero-dimer (**A**, **C**) and a hetero-tetramer (**B**, **D**) on the raft membrane are shown. The contact regions between the residues of amylin (red arrow) and those of tau (blue arrow) are identified in dashed rectangles in the average contact maps. The color-coded data points represent the average and standard deviation of contact between each amino acid residue in the oligomer from the last 5 μs of the CG simulation.

Table S1. Summary of ANOVA Analysis of protein effect on alpha-helix formation. The *F*- and *p*-values of the comparisons between the alpha-helical contents of two groups of protein aggregates for all raft surfaces are given. See the Abbreviations of the main text for the definitions of the protein groups.

**Analyses**	***F*-value**	***p*-value**
2tau vs. 1tam	23.07	0.00096**
4tau vs. 2tam	3.862	0.080936
1am′ vs. 1tam	27.16	0.00081**
2am′ vs. 2am	2.52	0.1468
1tau′ vs. 1tau	3.0245	0.116
2tau′ vs. 2tau	0.084	0.7802
1tam vs. 1tam+1tau	21.8558	0.001592*
2tam vs. 2am+2tau	2.213	0.1751

* $p < 0.01$
** $p < 0.001$
